# Bupropion Slow Release vs Placebo With Adaptive Incentives for Cocaine Use Disorder in Persons Receiving Methadone for Opioid Use Disorder: A Randomized Clinical Trial

**DOI:** 10.1001/jamanetworkopen.2023.2278

**Published:** 2023-03-15

**Authors:** Orrin D. Ware, Mary M. Sweeney, Colin Cunningham, Annie Umbricht, Maxine Stitzer, Kelly E. Dunn

**Affiliations:** 1 Johns Hopkins University School of Medicine, Baltimore, Maryland; 2 School of Social Work, University of North Carolina at Chapel Hill; 3 Now with National Institute of Mental Health, National Institutes of Health, Bethesda, Maryland; 4 Friends Research Institute, Baltimore, Maryland

## Abstract

**Importance:**

Opioid-stimulant co-use is a common problem with few evidence-based treatments.

**Objective:**

To examine bupropion slow release (SR) enhancement of a tailored abstinence incentive program for stimulant use in persons with opioid use disorder.

**Design, Setting, and Participants:**

This 26-week, double-blind, placebo-controlled randomized clinical trial with a 4-week follow-up period was conducted at 4 methadone treatment programs in Baltimore, Maryland. Included participants were persons receiving methadone for the treatment of opioid use disorder with past 3-month cocaine use and current cocaine use disorder between March 2015 and September 2019. Data were analyzed from November 2020 through August 2022.

**Interventions:**

A 6-week incentive induction period with monetary incentives for evidence of cocaine abstinence during thrice-weekly urine testing was conducted. Persons achieving 2 weeks of consecutive abstinence during induction were assigned to the relapse prevention group (20 individuals); otherwise, individuals were assigned to the abstinence initiation group (60 individuals). Participants were randomized within incentive groups to bupropion SR (150 mg oral twice daily; 40 participants) or placebo (40 participants). Incentives were available until week 26, and study medication ended week 30.

**Main Outcomes and Measures:**

The mean percentage of participants with cocaine abstinence (by negative urinalysis or self-report) during weeks 7 to 26 (ie, the incentive intervention period) and 27 to 30 (ie, the follow-up period) and the percentage of participants testing negative for cocaine at weeks 26 and 30 were assessed. Main effects of medication collapsed across incentive conditions and sensitivity analyses of medications within incentive conditions were assessed. Analyses were conducted in the modified intention-to-treat sample (ie, 80 individuals who received ≥1 dose of study medication) and completers (ie, 52 individuals who completed ≥1 visit during week 30).

**Results:**

Among 80 participants (42 Black [52.5% ] and 35 White [43.8%]; mean [SD] age, 45.7 (9.4) years; 52 males [65.0%]) receiving methadone for opioid use disorder, 40 participants were randomized to receive bupropion SR and 40 participants to receive placebo. No significant difference on urinalysis or self-reported cocaine use was observed between medication groups. Sensitivity analyses revealed differential patterns for incentive subgroups. Participants in the relapse prevention group had high abstinence (>80%; eg, during weeks 7-26 in the modified intention-to-treat analysis, 410 of 456 samples [89.9%] from participants in the bupropion SR group tested negative for cocaine) throughout the trial regardless of whether they were randomized to bupropion SR or placebo. Participants in the abstinence initiation group had better outcomes with bupropion SR than placebo throughout the trial (mean [SD] total number of samples testing negative for cocaine, 30.3 [21.6] samples for bupropion SR vs 17.1 [14.9] samples for placebo; *P* = .05) and more participants receiving bupropion SR than placebo were abstinent at the end of the study (20 of 30 participants [66.7%] vs 9 of 30 participants [30.0%]; *P* = .04).

**Conclusions and Relevance:**

In this randomized clinical trial, an overall benefit for bupropion SR vs placebo when combined with a financial abstinence incentive program was not observed. Results among incentive subgroups suggest that continued evaluation of medications, including bupropion SR, for stimulant treatment using a tailored approach that factors early abstinence into study design and interpretation may be needed.

**Trial Registration:**

ClinicalTrials.gov Identifier: NCT02111798

## Key Points

**Question:**

Compared with placebo, does bupropion slow release (SR) combined with contingent abstinence incentives improve abstinence from cocaine among individuals with cocaine use disorder who are receiving methadone treatment for opioid use disorder?

**Findings:**

In this randomized clinical trial of 80 individuals receiving methadone treatment for opioid use disorder, there was no significant overall effect of bupropion SR and financial incentives vs placebo and financial incentives for ongoing cocaine use.

**Meaning:**

These findings suggest that continued exploration of a tailored approach to examining treatments for stimulant use that factor early abstinence into the study design and data interpretation may be needed.

## Teaser

This randomized clinical trial compares the effect of abstinence incentives combined with either bupropion slow release or placebo on cocaine abstinence outcomes among individuals receiving methadone treatment for opioid use disorder.

## Introduction

Opioid and cocaine use are associated with morbidity and mortality, and co-use of opioids and stimulants is increasing in the United States.^1^ There are no criterion standard treatments for stimulant use disorder or opioid-stimulant co-use, which is particularly recalcitrant among persons receiving methadone for opioid use disorder (OUD) treatment.^2,3,4,5,6^ Bupropion slow release (SR) is a dual norepinephrine and dopamine reuptake inhibitor marketed for depression (Wellbutrin) and smoking cessation (Zyban) that has been associated with decreased methamphetamine use when coadministered with naltrexone.^7^ Mixed evidence supports bupropion for persons using cocaine while receiving methadone treatment.^8,9,10^ A study^11^ that layered bupropion SR with monetary incentives for urine-verified abstinence found that participants assigned to receive incentives and bupropion SR achieved greater cocaine abstinence than members of the control group.^12^ Pairing financial incentives with bupropion SR to slow or prevent relapse from occurring after incentive discontinuation may be a promising treatment strategy.

This double-blind, placebo-controlled randomized clinical trial examined whether bupropion SR and monetary-based incentives reduced cocaine use in persons maintained on methadone for OUD while accounting for the moderating role of early abstinence. Because persons who show early response to treatments (eg, abstinence) are highly likely to remain abstinent throughout treatment,^13^ early abstinence may be a potentially important clinical metric for determining treatment intensity^14^ that has not been examined for stimulant use disorder. Participants in this study received incentives for 6-weeks to determine early response patterns; persons who achieved abstinence received incentives to prevent relapse, while persons who did not achieve abstinence received enhanced incentives to promote abstinence. Following this lead-in period, participants were assigned via minimization to receive bupropion SR or placebo for the remainder of the trial. Primary outcomes were urinalysis and self-reported cocaine use over 30-weeks. The study hypothesized that bupropion SR compared with placebo and combined with tailored abstinence-responsive incentives would promote more initiation of cocaine abstinence in persons who did not initially achieve abstinence with incentives alone and would reduce relapse to cocaine use after incentive removal in persons who achieved early abstinence.

## Methods

The Johns Hopkins University Institutional Review Board approved this randomized clinical study, and all participants provided voluntary informed consent. This report follows the Consolidated Standards of Reporting Trials (CONSORT) reporting guideline for randomized trials, and the protocol is available in Supplement 1.

### Participants

**Figure 1.**
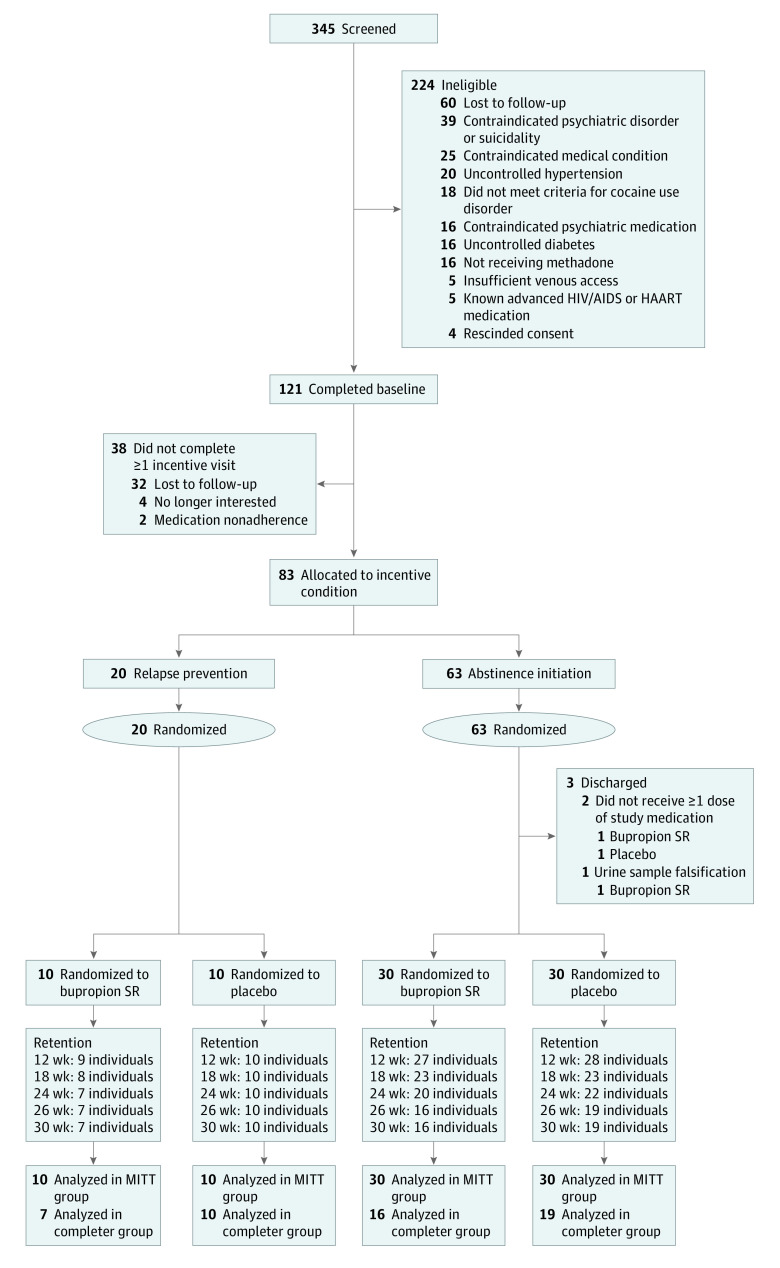
Study Flowchart Participants were assigned to incentive conditions based on abstinence achieved during the incentive induction period. Retention data reflect the percentage of participants completing all scheduled study assessments. HAART indicates highly active antiretroviral therapy; SR, slow release.

Participants were recruited from 4 methadone clinic programs in Baltimore, Maryland, between March 2015 and September 2019 using self and counselor referrals. Eligible participants were aged 18 years or older, were enrolled in methadone treatment, self-reported using cocaine 1 day or more in the past 30 days, provided a urine sample testing positive for cocaine at screening or in the past 3-month clinical record, and met *Diagnostic and Statistical Manual of Mental Disorders* (Fifth Edition) criteria for cocaine use disorder. Participants were excluded for evidence of pregnancy or contraindicated psychiatric conditions, medical conditions, and medications. Detailed eligibility criteria are provided in eTable 1 in Supplement 2. Participants self-reported race and ethnicity on a demographic measure designed to characterize the participant sample. Available categories for race were African-American, Asian, Caucasian, Native American, Pacific Islander, and more than 1 race. Overall, 345 individuals were screened and 121 were enrolled, among whom 38 participants (31.4%) left the trial before becoming eligible for randomization; 83 individuals were randomized to study medication, and 80 consumed 1 dose or more of medication and were included in the final modified intention-to-treat (MITT) analyses (Figure 1).

### Study Design

Eligible participants were enrolled in a 30-week randomized clinical trial with thrice-weekly visits. Participants completed a 6-week incentive lead-in period in which monetary incentives were available for evidence of urinalysis-verified cocaine abstinence beginning day 1. Participants who achieved 6 consecutive negative samples (ie, 2 weeks of abstinence) during this period were categorized to a relapse prevention (RP; 20 participants) incentive group. Participants who did not achieve 6 consecutive negative samples during weeks 1 through 6 were categorized to an abstinence initiation (AI; 60 participants) incentive condition. After assignment, participants were stratified separately within each incentive condition via a minimization procedure (Beck Depression Inventory summed total scores from screening; ≥14 vs <14; range, 0-63 and sex) to receive bupropion SR or placebo by the research pharmacist, who had no participant interaction. Participants continued to attend thrice-weekly visits for the duration of the 30-week protocol; urinalysis samples and self-reported cocaine use were collected at every visit. To facilitate data collection, participants received randomly allocated incentives (prize range, $0-$50) for session attendance independent of urinalysis outcomes.

### Incentive Conditions

Incentives were available during weeks 1 to 26 (eTable 2 in Supplement 2) for urinalysis-based evidence of cocaine abstinence. During weeks 1 to 6, incentives began at $0.50 per cocaine-negative sample and increased by $0.50 for each subsequent negative sample to a possible total of $9 per negative sample and a maximum week 1 through 6 earning of $85.50. After week 6, participants categorized into RP had payments increased to $11 for 12 weeks before they were tapered to $0 by week 26. After week 6, participants categorized into AI could earn $3, and values increased by $0.50 to $11 through week 26. Participants could earn bonuses ($3, $7, and $10) for 3 consecutive negative samples through week 26. Positive samples resulted in no earnings for that sample, reset the earning schedule to $0.50, and required 2 consecutive negative samples to return to the same place on the earning schedule. Total potential earnings were $675.00 for the RP and $725.50 for the AI groups. Participants were not told that incentive structures would change based on their initial treatment response.

### Study Medications

Overencapsulated, double-blind medication was ingested orally by participants twice daily during study weeks 2 to 30. Participants received double-blinded placebo (microcrystalline cellulose) until they met criteria for the RP or AI incentive schedules, at which time they were randomized to bupropion SR (40 participants) or placebo (40 participants). Participants assigned to bupropion SR were titrated from 150 mg to 300 mg over a 3-day period and maintained on 300 mg thereafter. Medication was dispensed weekly in blister packs.

### Study Outcomes

#### Urinalysis Testing for Cocaine

Urine samples were collected under same-sex staff observation and tested immediately onsite for the cocaine metabolite benzoylecgonine (150 ng/mL) using qualitative dipsticks. Urinalysis outcomes were the percentage of scheduled samples testing negative for cocaine, total number of negative samples, longest duration of continuous abstinence, and percentage of participants providing 3 negative samples during week 26 (ie, the end of intervention) and week 30 (ie, the end of study). Data were analyzed separately during weeks 7 to 26 (active incentives), 27 to 30 (after incentives), and week 30 (final evaluative period).

#### Self-reported Cocaine Use

Participants completed a weekly timeline follow-back procedure with staff to measure self-reported cocaine use. This use was operationalized as yes or no independent of route or quantity and analyzed as percentage negative reports during weeks 7 to 26 and 27 to 30.

#### Medication Compliance

Point-prevalence compliance was assessed via semiquantitative levels of bupropion and its metabolite hydroxybupropion in urine samples. These were collected during weeks 12, 18, 24, and 30.

#### Retention

Retention was quantitatively assessed. It was evaluated as the number of scheduled urine samples submitted and percentage of participants attending 1 or more visits during week 30.

#### Study Satisfaction

Adverse events (AEs) were assessed at each study visit and classified by severity (mild, moderate, or severe) and study relationship (unrelated and possibly, probably, and definitely related). Participants also reported what medication (placebo or bupropion SR) they thought they had received, how useful the medication and incentives were (score range, 0-10) for cocaine abstinence, and whether they would request the medication from their doctor (yes or no).

### Statistical Analysis

Power analyses initially conducted independently for each incentive condition (RP and AI) indicated that a sample of 50 participants per medication group per incentive condition was necessary (for a total of 200 participants). Owing to recruitment challenges, a revised power analysis was conducted that collapsed across incentive conditions to evaluate bupropion SR vs placebo, yielding a total of 50 participants per medication group while covarying for incentive conditions. Recruitment ended early due to the SARS-CoV-2 pandemic, with 83 of 100 enrolled participants randomized (83.0%) and 80 participants included in the MITT analyses (80.0%).

Demographic and drug use characteristics were compared across groups using χ^2^ for dichotomous and independent groups *t* tests for continuous variables. Main effects of the randomized condition (study medication) were assessed by collapsing across incentive conditions and including incentive schedule (RP or AI) as a covariate in analyses. Outcomes were evaluated as a function of medication and time during the active incentive (weeks 7-26) and postincentive (weeks 27-30) periods. Urine qualitative results and self-reported cocaine use were assessed using generalized estimating equations, and total number of negative samples and longest duration of continuous abstinence were assessed using mixed models that treated missing data as missing (missing-missing) and as positive (missing-positive) for cocaine use; absences that were excused in advance were not documented as missing. Retention, medication compliance, percentage of participants testing abstinent (missing-positive only), and AEs were compared within medication and incentive groups using χ^2^ or independent group *t* tests. Given the significance of the incentive condition throughout analyses, sensitivity analyses within each incentive condition to evaluate the main effects of medication and time within incentive conditions were conducted. Analyses were completed within MITT (defined as consuming ≥1 dose of study medication) and completer (≥1 visit during week 30; 52 participants) groups, and methadone clinic was included as a covariate for all analyses. We conducted analyses using SAS statistical software version 9 (SAS Institute) and SPSS statistical software version 28 (IBM); 2-sided α was set at .05, with no adjustments for multiple comparisons. Data were analyzed from November 2020 through August 2022.

## Results

### Participants

Among 80 participants (1 American Indian [1.3%], 42 Black [52.5%], 35 White [43.8%], and 2 with more than 1 race [2.5%]; 3 Hispanic [3.8%]; mean [SD] age, 45.6 (9.4) years; and 52 males [65.0%]), 40 participants were randomized to receive bupropion SR and 40 participants to receive placebo. Between-group comparisons are presented in eTable 3 in Supplement 2. No demographic differences were observed between medication groups. Participants in the RP group were less likely than those assigned to AI to provide a cocaine-positive urine sample on study day 1 (7 participants [35.0%] vs 52 participants [86.7%]; χ^2^_1_ = 20.7; *P* < .001) but met more criteria for alcohol use disorder (mean [SD] 1.5 [3.0] criteria vs 0.5 [1.6] criteria; *t*_78_ = 2.0; *P* = .05). Participants in the RP group also earned significantly more mean (SD) incentives than those in the AI group during the trial ($544.42 [$182.30] vs $127.90 [$196.91]; *t*_71_ = −7.93; *P* < .001). Persons who left prior to randomization (38 participants) were significantly more likely to be female (21 participants [55.3%] vs 28 participants [35.0%]; *P* = .03) and maintained on a lower methadone dose (mean [SD], 75.3 [29.2] mg vs 87.7 [24.3] mg; *P* = .02) than randomized participants.

### Urinalysis Outcomes

**Figure 2.**
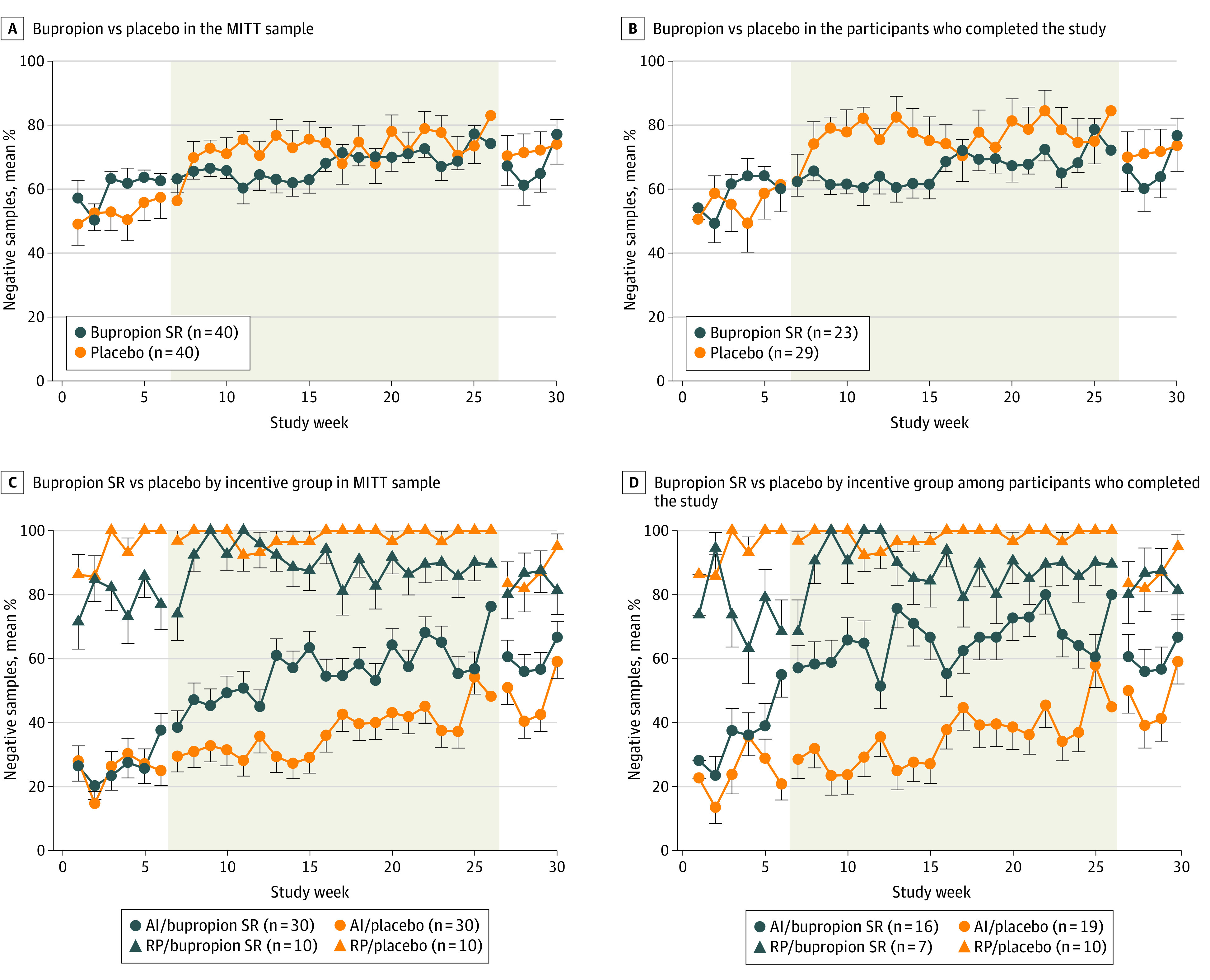
Urinalysis Results Over Time Data represent the mean percentage (standard error of the mean) of urine samples tested immediately onsite for evidence of cocaine use as a function of study week (range, weeks 0-30), with missing data treated as missing. Time periods represent weeks 1 to 6 (double-blind placebo lead-in period), 7 to 26 (active pharmacotherapy with incentives), and 27 to 30 (pharmacotherapy-only follow-up period). Top panels present data for bupropion slow release (SR) and placebo among A, the modified intention-to-treat (MITT; defined as ≥1 dose of study medication) and B, completer (defined as being present during the final study week) samples. Urine results as a function of incentive groups abstinence initiation (AI) and relapse prevention (RP) are presented within each medication condition for C, MITT and D, completer samples.

**Figure 3.**
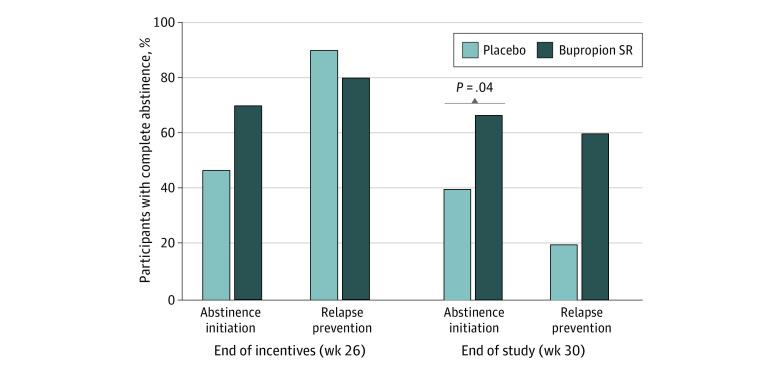
Percentage of Participants Testing Negative for Cocaine Use Percentages are presented of participants who provided 3 urine samples that tested negative for cocaine at A, end of the incentive intervention (week 26) and B, end of the study (week 30) as a function of being in the abstinence incentive condition and receiving bupropion slow release (SR) (30 participants) or placebo (30 participants) or the relapse prevention condition and receiving bupropion SR (10 participants) or placebo (10 participants).

No significant main effects of study medication, time, or time × medication interactions were observed for urinalysis-verified cocaine abstinence when collapsed across incentive conditions for missing-missing or missing-positive analyses. This was true during active incentive (weeks 7-26) and postincentive (weeks 27-30) periods within MITT and completer analyses (Figure 2A-B). Completers in both medication groups demonstrated higher rates of abstinence compared with the MITT group (Table 1, Figure 2). Significantly more participants in the bupropion SR group achieved complete abstinence during week 30 than those in the placebo group (Figure 3; 26 participants [65.0%] vs 14 participants [35.0%]; χ^2^_1_ = 7.2; *P* = .007), although differences were not significant during week 26 (26 participants [65.0%] vs 23 participants [57.5%]; *P* = .12).

**Table 1.** Primary Urinalysis and Self-reported Outcomes

Analysis group^a^	Samples, No./total No. (%)		*P* value
	Bupropion SR	Placebo	
**MITT**			
Participants, No.	40	40	NA
Urinalysis-verified abstinence			
Samples testing negative for cocaine, weeks 7-26			
Missing-missing	1005/1542 (65.2)	970/1687 (57.5)	.33
Missing-positive	1005/2400 (41.9)	970/2400 (40.4)	.70
Samples testing negative for cocaine, weeks 27-30			
Missing-missing	125/183 (68.3)	169/280 (60.4)	.65
Missing-positive	125/480 (26.0)	169/480 (35.2)	.24
Total No. negative samples, mean (SD)			
Weeks 7-26 (range, 0-72)^b^	28.3 (25.8)	28.5 (25.4)	.72
Weeks 27-30 (range, 0-28)^b^	3.1 (0.4)	4.2 (4.1)	.26
Longest-duration continuous abstinence, weeks 7-26 (range, 0-72), mean (SD)^b^	14.4 (18.2)	14.1 (18.3)	.88
Self-reported abstinence			
Total No. negative samples, mean (SD)			
Weeks 7-26	85.9 (48.1)	96.1 (47.7)	.39
Weeks 27-30	11.0 (11.9)	15.3 (10.8)	.11
Abstinence weeks 7-26			
Missing-missing	3436/4343 (79.1)	3844/4754 (80.9)	.92
Missing-positive	3436/5600 (61.4)	3844/5600 (68.6)	.44
Abstinence weeks 27-30			
Missing-missing	440/534 (82.4)	610/696 (87.6)	.74
Missing-positive	440/1120 (39.3)	610/1120 (54.5)	.09
**Completer**			
Participants, No.	29	23	NA
Urinalysis-verified abstinence			
Samples testing negative for cocaine, weeks 7-26			
Missing-missing	834/1130(73.8)	884/1482 (59.6)	.12
Missing-positive	834/1380 (60.4)	884/1740 (50.8)	.16
Samples testing negative for cocaine, weeks 27-30			
Missing-missing	125/183 (68.3)	166/277 (59.9)	.18
Missing-positive	125/276 (45.3)	166/348 (47.7)	.25
Total No. negative samples, mean (SD)			
Weeks 7-26 (range, 0-72)^b^	36.3 (21.4)	30.5 (22.4)	.18
Weeks 27-30 (range, 0-28)^b^	5.4 (4.6)	5.7 (3.9)	.77
Longest duration continuous abstinence weeks 7-26 (range, 0-72), mean (SD)^b^	21.0 (20.7)	18.1 (19.7)	.45
Self-reported abstinence			
Total No. negative samples, mean (SD)			
Weeks 7-26	115.4 (33.1)	117.0 (29.0)	.78
Weeks 27-30	19.1 (9.3)	21.0 (6.1)	.45
Abstinence weeks 7-26			
Missing-missing	1900/2179 (87.2)	2934/3607 (81.3)	.39
Missing-positive	1900/2940 (64.6)	2934/4340 (67.6)	.72
Abstinence weeks 27-30			
Missing-missing	261/294 (88.8)	444/495 (89.7)	.31
Missing-positive	261/588 (44.4)	444/868 (51.2)	.47

Abbreviations: MITT, modified intention to treat; NA, not applicable; SR, slow release.

^a^ The MITT population (80 participants) was defined as randomized participants taking 1 or more doses of study medication. The completer population (52 participants) was defined as participants providing a urine sample during the final study week (week 30). Data are separated by postminimization active intervention (weeks 7-26) and postintervention follow-up (weeks 27-30) periods.

^b^ Ranges are the total number of urine samples available per participant.

### Self-report Outcomes

Consistent with urinalysis results, there were no observed effects of study medication, time, or time × medication interactions on self-reported cocaine use. This was true when collapsed across incentive conditions for MITT and completer samples, for missing-missing and missing-positive analyses, and for data collected during weeks 7 through 26 or 27 through 30 (Table 1). For example, in the MITT population, 1005 of 1542 samples (65.2%) in the bupropion SR group and 970 of 1687 samples (57.5%) in the placebo group tested negative for cocaine in weeks 7 through 26 (*P* = .33).

### Medication Compliance

Significant main effects of medication were detected on quantitative levels of bupropion (*F*_1,40_ = 12.8; *P* < .001) and hydroxybupropion (*F*_1,40_ = 9.6; *P* = .01) during weeks 12, 18, and 24, suggesting participant adherence with study medication. Although bupropion levels did not differ between groups at week 30, hydroxybupropion levels remained significantly higher in the bupropion SR vs placebo group (*F*_1,36_ = 4.4; *P* = .04).

### Retention and Urine Submissions

Of 5760 scheduled samples, 3917 samples (68.0%) were submitted. When collapsed across incentive conditions, there were no significant differences between participants receiving placebo vs those receiving bupropion SR in the percentage of urine samples submitted (2059 of 2880 samples [71.5%] vs 1854 of 2880 samples [64.4%]; *t*_78_ = 1.15; *P* = .25) or participants who completed 1 or more visits during week 30 (23 participants [57.5%] vs 17 participants [42.5%]; χ^2^_1_ = 1.98; *P* = .12). Participants in the RP group were more likely to submit samples (29 participants [72.5%] vs 23 participants [57.5%]; *t*_78_ = −3.47; *P* < .001) and complete 1 or more visits during week 30 (32 participants [85.0%] vs 23 participants [58.3%]; χ^2^_1_ = 4.97; *P* = .02) than participants in the AI group. Although interactions were not significant, the RP bupropion SR group had more missing samples than the RP placebo group during weeks 27 to 30.

### Study Satisfaction

A total of 18 participants in the MITT population (22.5%) experienced a possible study-related AE (eTable 4 in Supplement 2). There was no significant difference by medication assignment (placebo vs bupropion SR) in the number of participants who experienced an AE (14 participants [17.5%] vs 22 participants [27.5%]; χ^2^_1_ = 1.15; *P* = .21) or mean (SD) number of AEs (2.75 [0.75] AEs vs 4.75 [0.99] AEs); *t*_78_ = −1.02; *P* = .31). Among 30 reported events, 20 AEs were classified as mild (66.7%), 7 AEs as moderate (23.3%), and 3 AEs as severe (10.0%). There were 17 serious AEs reported; all were associated with hospitalization for preexisting medical conditions and deemed unrelated to study participation.

Blinding was maintained, with 14 participants in the placebo (46.7%) and 11 participants in the bupropion SR (37.9%) groups correctly guessing their assignments. Participants gave a lower mean (SD) usefulness rating of study medication (3.8 [3.7]) than incentives (6.9 [3.1]); however placebo and bupropion SR groups rated their medication similarly (mean [SD] rating, 3.9 [3.7] vs 2.9 [3.4];* t*_54_ = 1.01; *P* = .32); with 48 participants (60.0%) and 28 participants (54.1%) stating that they would request the medication from their doctor (χ^2^_1_ = 0.17; *P* = .45), respectively. The mean (SD) ratings for incentives were significantly higher for participants in the RP vs AI groups (8.1 [2.4] vs 6.4 [3.2]; *t*_50_ = 1.85; *P* = .02).

### Sensitivity Analyses Within Incentive Groups

#### Relapse Prevention

Among 20 participants assigned to the RP group, 10 participants received bupropion SR and 10 participants received the placebo. Persons assigned to RP achieved almost complete cocaine abstinence during weeks 1 to 6, and abstinence was largely sustained throughout the study (Table 2, Figure 2). Although bupropion SR was associated with less abstinence compared with placebo during weeks 7 to 26, outcomes were largely influenced by missing data. In the MITT missing-missing analysis, 410 of 456 samples (89.9%) and 559 of 569 samples (98.2%) from participants in the bupropion SR and placebo groups, respectively, tested negative for cocaine. When missing data (due almost exclusively to 3 participants who left treatment) were imputed as positive, abstinence in persons receiving bupropion SR decreased from 89.9% to 410 of 600 samples (68.3%; χ^2^_1_ = 4.5; odds ratio, 0.40; 95% CI, 0.11-1.42; *P* = .03). However, there was no longer a between-group effect of medication when tested only among completers, with negative tests for cocaine among 349 of 420 missing-positive samples (83.1%) and 349 of 394 of missing-missing samples (88.6%) for the bupropion group and 559 of 600 missing-positive samples (93.2%) and 559 of 569 missing-missing samples (98.2%) for the placebo group. The total number of samples testing negative during weeks 7 to 26 was also statistically significantly different between bupropion SR and placebo groups (410 of 600 samples [68.3%] vs 559 of 600 samples [93.2]; χ^2^_2_ = 4.42; *P* = .04) (Table 2). Self-reported rates of abstinence for weeks 7 to 26 were 1058 of 1093 missing-missing samples (96.8%) among participants receiving bupropion SR and 1362 of 1400 missing-missing samples (97.3%) among those receiving placebo.

**Table 2.** Primary Urinalysis and Self-reported Outcomes Within Incentive Conditions

Analysis group^a^	AI samples, No./total No. (%)		*P* value^b^	RP samples, No./total No. (%)		*P* value^b^
	Bupropion SR	Placebo		Bupropion SR	Placebo	
**MITT**						
Participants, No.	30	30	NA	10	10	NA
Urinalysis-verified abstinence						
Samples testing negative for cocaine, weeks 7-26						
Missing-missing	595/1086 (54.8)	411/1118 (36.8)	.17	410/456 (89.9)	559/569 (98.2)	NA
Missing-positive	595/1800 (33.1)	411/1800 (22.8)	.16	410/600 (68.3)	559/600 (93.2)	.04
Samples testing negative for cocaine, weeks 27-30						
Missing-missing	73/121 (60.3)	92/191 (48.2)	.56	52/62 (83.9)	77/89 (86.5)	.23
Missing-positive	73/360 (20.3)	92/360 (25.6)	.49	52/120 (43.3)	77/120 (64.2)	.28
Total No. negative samples, mean (SD)						
Weeks 7-26 (range, 0-72)^c^	19.8 (20.3)	13.7 (15.2)	.16	41.0 (20.3)	55.9 (4.98)	.06
Weeks 27-30 (range, 0-28)^c^	2.4 (2.8)	3.1 (3.5)	.51	5.2 (5.5)	7.7 (4.1)	.35
Longest duration continuous abstinence weeks 7-26 (range, 0-72), mean (SD)^c^	10.4 (16.4)	5.7 (9.3)	.92	26.3 (19.1)	39.2 (15.3)	.11
Self-reported abstinence						
Total No. negative samples, mean (SD)						
Weeks 7-26	79.3 (47.4)	82.7 (47.8)	.82	105.8 (46.8)	136.2 (10.0)	.08
Weeks 27-30	10.5 (12.0)	12.0 (10.5)	.67	12.4 (11.9)	25.0 (3.4)	.01
Abstinence weeks 7-26						
Missing-missing	2378/3250 (73.2)	2482/3354 (74.0)	.90	1058/1093 (96.8)	1362/1400 (97.3)	NA
Missing-positive	2378/4200 (56.6)	2482/4200 (59.1)	.80	1058/1400 (75.6)	1362/1400 (97.3)	NA
Abstinence weeks 27-30						
Missing-missing	316/397 (79.6)	360/439 (82.0)	.96	124/137 (90.5)	250/257 (97.3)	NA
Missing-positive	316/840 (37.6)	360/840 (42.9)	.65	124/280 (44.3)	250/280 (89.3)	NA
**Completer**						
Participants, No.	16	19	NA	7	10	NA
Urinalysis-verified abstinence						
Samples testing negative for cocaine, weeks 7-26						
Missing-missing	481/732 (65.7)	325/913 (35.6)	.04	349/394 (88.6)	559/569 (98.2)	.23
Missing-positive	485/960 (50.1)	325/1140 (28.5)	.04	349/420 (83.1)	559/600 (93.2)	.27
Samples testing negative for cocaine, weeks 27-30						
Missing-missing	73/121 (60.3)	89/188 (47.3)	.44	52/62 (83.9)	77/89 (86.5)	.73
Missing-positive	73/192 (38.0)	89/228 (39.0)	.68	52/84 (61.9)	77/120 (64.2)	.88
Total No. negative samples, mean (SD)						
Weeks 7-26 (range, 0-72)^c^	30.3 (21.6)	17.1 (14.9)	.05	49.9 (14.2)	55.9 (5.0)	.33
Weeks 27-30 (range, 0-28)^c^	4.6 (4.3)	4.7 (3.4)	.93	7.4 (5.1)	7.7 (4.1)	.72
Longest duration continuous abstinence weeks 7-26 (range, 0-72), mean (SD)^c^	11.1 (14.2)	4.5 (5.9)	.09	32.0 (19.0)	39.2 (15.3)	.10
Self-reported abstinence						
Total No. negative samples, mean (SD)						
Weeks 7-26	108.9 (36.8)	106.9 (30.8)	.59	130.3 (15.7)	136.2 (10.0)	.45
Weeks 27-30	19.8 (9.3)	18.9 (6.3)	.68	17.7 (10.0)	25.0 (3.4)	.11
Abstinence weeks 7-26						
Missing-missing	1175/1423 (82.6)	1847/2487 (74.3)	.40	725/756 (95.9)	1087/1120 (97.1)	NA
Missing-positive	1175/1960 (59.9)	1847/3220 (57.4)	.94	725/980 (74.0)	1087/1120 (97.1)	NA
Abstinence weeks 27-30						
Missing-missing	161/181 (89.0)	242/291 (83.2)	.57	100/113 (88.5)	202/204 (99.0)	NA
Missing-positive	161/392 (41.1)	242/644 (37.6)	.77	100/196 (51.0)	202/224 (90.2)	NA

Abbreviations: AI, abstinence initiation; MITT, modified intention to treat; NA, not applicable; RP, relapse prevention.

^a^ The MITT population (80 participants) was defined as randomized participants taking 1 or more doses of study medication. The completer population (52 participants) was defined as participants providing a urine sample during the final study week (week 30).

^b^ *P* values represent main effect of group within each incentive condition. NA indicates that the statistical model converged.

^c^ Ranges are the total number of urine samples available per participant.

#### Abstinence Initiation

Among 60 participants assigned to the AI group, 30 participants received bupropion SR and 30 participants received placebo. In contrast, bupropion SR was associated with increased abstinence compared with placebo in persons assigned to AI (Table 2, Figure 2). In the MITT analysis, rates of cocaine-negative urine samples for persons receiving bupropion SR (595 of 1800 missing-positive samples [33.1%] and 595 of 1086 missing-missing samples [54.8%]) compared with those receiving placebo (411 of 1800 missing-positive samples [22.8%] and 411 of 1118 missing-missing samples [36.8%]) were not significantly different for the missing-missing (*P* = .17) or missing-positive (*P* = .16) analyses during weeks 7 to 26. When analyzed within completers, bupropion SR was associated with significantly higher rates of urinalysis-verified abstinence than placebo in the missing-missing (481 of 732 samples [65.7%] vs 325 of 913 samples [35.6%]; *P* = .04) and missing-positive (485 of 960 samples [50.1%] vs 325 of 1140 samples [28.5%]; *P* = .04) analyses during weeks 7 to 26 but not weeks 27 to 30. The mean (SD) total number of samples testing negative for cocaine was also significantly higher among completers who were assigned to bupropion SR vs placebo (30.3 [21.6] samples vs 17.1 [14.9] samples; *P* = .05). More participants in the AI group who received bupropion SR vs placebo achieved complete abstinence during week 30 (20 participants [66.7%] vs 12 participants [40.0%]; χ^2^_1_ = 4.29; *P* = .04), and abstinence was not significantly different between groups during week 26 (21 participants [70.0%] vs 14 participants [46.7%]; *P* = .06) (Figure 3). Self-reported use did not differ significantly between groups at weeks 7 to 26 (2378 of 3250 missing-missing samples [73.2%] among participants receiving bupropion SR and 2482 of 3354 missing-missing samples [74.0%] among those receiving placebo).

## Discussion

This double-blind, randomized clinical trial compared bupropion SR with placebo as a treatment for cocaine use in persons receiving methadone as treatment for OUD. Incentive delivery was tailored to participant early incentive response during the 6-week double-blind lead-in period. Among enrolled participants, 31% of participants left the trial before becoming eligible for randomization. MITT analyses showed no overarching benefit of bupropion SR compared with placebo when collapsed across incentive conditions. Sensitivity analyses showed that persons who achieved early abstinence (RP group) did not benefit from concurrent bupropion SR treatment but that persons who continued using cocaine after incentives began (AI group) achieved higher levels of abstinence when receiving bupropion SR with incentives vs placebo with incentives. Bupropion SR also produced a persistent effect on abstinence after incentive removal in participants in the AI group. In contrast, participants who were responsive to incentives early in treatment (RP group) did not demonstrate differential abstinence as a function of medication assignment once incentives were tapered, although this may be related to the low rate of cocaine use in that group overall. Together, these data may support further investigations into pharmacotherapy in the context of tailored incentives.

This trial was unique in its assessment of combined incentive and pharmacotherapy treatment as a function of early treatment response. Despite evidence that early treatment response was associated with future abstinence,^13^ pharmacotherapy evaluations for cocaine use disorder do not routinely factor early abstinence in their methods or analyses. That participants in the RP group in this trial maintained near-complete abstinence throughout the study independent of medication and incentives and the lack of treatment signal in the RP but not AI group may support factoring early abstinence into stimulant treatment trials. Inclusion of participants in the RP group in the overall analyses may have obscured the significant benefit bupropion SR conferred on persons assigned to AI. This finding may also help explain variability observed in prior examinations of bupropion SR for cocaine use.^8,9,12^

Bupropion SR may enhance the effects of financial incentives in persons who were unable to achieve early abstinence with incentives alone. The percentage of participants who received bupropion SR and achieved complete abstinence during weeks 26 and 30 overall (65% at both time points) was greater than the highest methamphetamine abstinence produced in a prior examination of bupropion SR and naltrexone (16.5%).^7^ The degree to which outcomes were influenced by a self-selection bias (eg, owing to the 31% of eligible participants who dropped out during the 6-week induction period) remains uncertain and should be considered in future evaluations of combined treatments.

### Limitations

This study has several limitations. One limitation to these data are that outcomes were highly influenced by missing data, which was not unexpected given the extended (30-week) monitoring period and thrice-weekly visit schedule. Missingness within the RP MITT subgroup contributed to a visible advantage of placebo compared with bupropion SR in missing-positive analyses, although missing data in this group appeared to be unrelated to study medication or procedures. In persons assigned to AI, overall rates of urine sample submission declined at a similar rate within both medication groups during the 7 to 26–week incentive intervention period. The positive signal for bupropion SR among participants in the AI group was not significant in the MITT sample but was statistically-significant in the completer subsample. Although the completer sample had less variability and missing data than the MITT sample, it was also a self-selected sample that was therefore subject to bias, which complicates interpretation. The lack of data on concurrent opioid use by study participants and changes in methadone medication treatment is a limitation of this study. Additionally, several between-group comparisons may not have reached statistical significance owing to lack of power but may still represent clinically meaningful differences.

## Conclusions

This randomized clinical trial failed to support an overarching benefit of bupropion SR compared with placebo when combined with a financial abstinence incentive program. However, differential effects were observed for participants who did and did not achieve early abstinence during an incentive-only study phase, with those who failed to achieve early abstinence benefitting from bupropion SR along with continued financial abstinence incentives. Data suggest that continued examination of bupropion SR with incentives for cocaine abstinence may be warranted and that trials treating stimulant use should account for early abstinence.

## Notes

Supplement 1.
Trial Protocol and Statistical Analysis Plan

Supplement 2. **eTable 1.** Study Inclusion Criteria **eTable 2.** Incentive Structure **eTable 3.** MITT Participant Demographics **eTable 4.** Adverse Events

Supplement 3.
Data Sharing Statement
