# Supplementary material for: Bupropion Slow Release vs Placebo With Adaptive Incentives for Cocaine Use Disorder in Persons Receiving Methadone for Opioid Use Disorder: A Randomized Clinical Trial
Source: JAMA Netw Open. 2023 Mar 15;6(3):e232278. doi: 10.1001/jamanetworkopen.2023.2278 (PMC10018324; doi:10.1001/jamanetworkopen.2023.2278)
Supplement: Supplement 1. — Trial Protocol and Statistical Analysis Plan [file jamanetwopen-e232278-s001.pdf]

Official Title: Medication Enhanced CM for Cocaine Dependence

NCA Number: 02111798

Document Date: January 2, 2019

Date: January 2, 2019
Principal Investigator: Kelly E. Dunn, Ph.D.
Application Number: NA00090062

JHM IRB - eForm A – Protocol

- **Use the section headings to write the JHM IRB eForm A, inserting the appropriate material in each. If a section is not applicable, leave heading in and insert N/A.**
- **When submitting JHM IRB eForm A (new or revised), enter the date submitted to the field at the top of JHM IRB eForm A.**

****

1. Abstract

Provide no more than a one page research abstract briefly stating the problem, the research hypothesis, and the importance of the research.

The efficacy of behavior therapies may be enhanced by certain medications, particularly those that act on dopaminergic systems. This project will examine effects of bupropion on initiation and maintenance of cocaine abstinence in a population of cocaine dependent methadone maintenance patients. Bupropion appears to be the most promising medication for this purpose because of its previously demonstrated efficacy and safety as well as its pharmacological actions at dopamine systems.

Methadone maintained cocaine dependent study participants (N = 100) will be stratified according to whether or not they stop their cocaine use during initial exposure to an abstinence incentive (contingency management) procedure and will be randomly assigned to receive bupropion XL (300mg/day) versus placebo for the remainder of the study. Our hypothesis is that bupropion as compared to placebo treatment will both enhance rates of abstinence initiation in those who have difficulty stopping cocaine and retard rates of relapse in those who initially stopped their cocaine use.

Participants will receive a CM schedule that is tailored to their initial CM response and outcomes will be tracked over a 6-month time frame that includes assessment of drug use outcomes after incentives have stopped. In addition, the project will provide novel information about mechanisms of medication effects by measuring subjective drug effects, drug vs money choices, and self-reports of pleasure derived from daily non-drug activities. We hypothesize that bupropion compared to placebo participants will report non-drug activities as more pleasurable (reinforcing), will engage in more of them and that measures of non-drug reinforcement will mediate abstinence outcomes.

Overall, this research will provide new and valuable information about combined behavioral-pharmacological treatments and specifically the conditions under which medication may enhance effects of CM. If hypothesized synergies can be demonstrated, the study will point the way to a significant advance in improved treatment outcomes for this critical group of drug abusers. The proposed study is compelling because it conceptually differentiates the two key clinical issues in treatment of stimulant abusers- abstinence initiation and relapse prevention. It uses a design that efficiently and effectively tests a combined treatment approach for each clinical issue and as well examines cognitive function and reinforcement-based mediators. The research will add to understanding of the interplay between brain reinforcement systems and drug-seeking behavior. Finally, it will make an important contribution to behavioral therapy development by exploring a novel solution to limitations previously noted for CM that include lack of response in some patients and relapse after withdrawal of incentives.

2. Objectives (include all primary and secondary objectives)

Specific aims of the project are the following:

a. Examine in those who initially achieve abstinence with CM the effects of bupropion XL (300 mg/day) versus placebo on 24-week abstinence outcomes during and after CM.
   Hypothesis: Among participants who achieve initial abstinence, those treated with bupropion compared to placebo will have significantly longer delays to resumption of drug use and higher rates of abstinence at 24-weeks.

b. Examine in those who initially fail to achieve abstinence with CM the effects of active bupropion XL (300 mg/day) versus placebo medication on subsequent abstinence initiation outcomes.
   Hypothesis: Among participants who failed to achieve initial abstinence, those treated with bupropion vs placebo will have significantly higher rates of abstinence at 12 and 24 weeks.

c. Identify mechanisms of bupropion-enhanced outcomes by administering self-report instruments that assess response to non-drug reinforcement.
   Hypothesis1: Bupropion compared to placebo participants will report both money and non-drug activities as more reinforcing and will engage in more of the latter.
   Hypothesis 2: Enhanced non-drug reinforcement will mediate abstinence outcomes

d. Determine whether response to abstinence-contingent reinforcers can be predicted by pre-treatment response to measures of drug vs non-drug relative reinforcing efficacy.

e. Explore the effects of bupropion XL (300 mg) vs placebo on secondary outcome measures of cognition, impulsivity, HIV risk behaviors and tobacco smoking.
   Hypothesis: Bupropion compared to placebo will improve attention, short-term memory and other cognitive processes and may decrease risky behaviors and tobacco smoking.

3. Background (briefly describe pre-clinical and clinical data, current experience with procedures, drug or device, and any other relevant information to justify the research)

There are few efficacious treatments for stimulant abuse, but those that have been identified are behavior therapies. In particular, the most effective behavior therapy approach available arguably involves the addition to counseling of contingency management procedures in which tangible incentives are offered for evidence of sustained abstinence from stimulant drugs (e.g. Dutra et al., 2008). On a background of extensive efficacy testing, two large clinical trials conducted in the CTN verified the effectiveness of this approach when applied with stimulant users enrolled in either methadone maintenance (Peirce et al., 2006) or psychosocial counseling (Petry et al., 2005) treatments. There are nonetheless two areas in which abstinence incentive therapies fall short. One is that only about half or fewer of the drug users exposed to these interventions respond by initiating prolonged periods of abstinence (Silverman et al., 1996; 2004). Secondly, many who do stop use during incentive interventions return to drug use after withdrawal of the interventions (Silverman et al., 1996; 2004). Methods that could counter these shortfalls and further improve treatment outcome for stimulant users would be very valuable.

There is also a growing body of evidence suggesting that certain medications can boost the efficacy of abstinence-focused contingency management. One of the first studies reported was by Kosten and colleagues (Kosten et al., 2003) who randomly assigned 160 buprenorphine-maintained cocaine abusers to receive desipramine (150mg/day) or placebo crossed with abstinence-contingent versus non-contingent voucher reinforcement (maximum earnings of $738 over 12 weeks). A significant interaction was observed. Overall percent of opiate and cocaine-free urines during weeks 3-12 of the intervention was 50% for the combined therapy group versus 25-29% for each of the other groups, with no significant differences between control and single therapy conditions. This was a convincing demonstration of a medication-CM interaction, especially since the CM did not appear to have any beneficial effects on its own Another convincing study was published by Schmitz and colleagues (Schmitz et al., 2008) who

randomly assigned 161 cocaine dependent patients to receive levodopa/carbidopa (400/100mg bid) crossed with one of 3 behavior therapy interventions (clinical management, cognitive-behavior therapy or voucher-based abstinence reinforcement; maximum earnings of approximately $1000 over 12 weeks). As in the Kosten et al. study, the largest intervention effect was seen with the group that received the medication combined with abstinence incentives. Further, the abstinence incentive condition was the only behavior therapy condition in which a significant effect of medication versus placebo was observed in planned post-hoc testing. Specifically, 60% versus 23% cocaine negative urines were delivered by participants in the levodopa/carbidopa vs placebo conditions when background therapy was abstinence-contingent CM. Although levo-dopa is not especially practical for clinical use, it has precise pharmacological specificity for enhancing dopaminergic neurotransmission. Thus, Schmitz et al (2008) were able to put forth an hypothesis and convincing argument that the mechanism of the interaction was likely enhanced response to rewarding stimuli (in this case, the CM intervention).

Bupropion is another anti-depressant medication that works in part by enhancing dopamine neurotransmission. In several prior trials, this medication did not show benefits when given on its own with a psychosocial counseling therapy as background (Elkashof et al., 2008; Margolin et al., 1995; Shoptaw et al., 2008a,b), although a recent re-analysis of the Elkashof study (McCann & Li, 2012) with methamphetamine abusers receiving CBT as a background therapy used a novel analysis approach to show that bupropion produced longer durations of abstinence among those who initiated abstinence (i.e. a relapse prevention or relapse delay effect). Bupropion in two studies has had a synergistic effect in combination with abstinence incentive interventions. Poling and colleagues (Poling et al., 2006) randomly assigned 106 methadone maintained cocaine users to receive bupropion (300mg/day) or placebo crossed with a behavior therapy that did or did not reinforce opiate and cocaine-free urine samples (maximum earnings of $462 over 12 weeks). Two outcome measures were significantly different for CM bupropion vs CM placebo groups: consecutive weeks of cocaine abstinence (6.7 CMB vs 4.3 CMP) and probability of a cocaine positive sample in weeks 24-25 (.33 CMB vs .57 CMB). This drug vs placebo difference is impressive considering the relatively small sample sizes studied and the multiple behavioral targets for the CM intervention (opiates, cocaine and abstinence-related goal activities) that could have diluted effects of CM on cocaine use specifically. Additional evidence of the apparent synergy between contingent incentives and bupropion comes from a smoking cessation study recently reported by Gray et al. (2010) in which adolescents who received bupropion (300 mg/day) plus abstinence incentives ($275 available over a 6-week program) had a 27% smoking abstinence rate at end of treatment compared with abstinence rates of 8-10% for groups that received either of the treatments alone or no treatment.

While pharmacological specificity of the interaction has not been explicitly researched, it is notable that one published study using tryptophan (Jones et al., 2004) another using the glutaminergic antagonist memantine (Bisaga et al., 2010) and an unpublished study using topiramate (Umbricht, personal communication) have failed to find an advantage for the combination of medication and abstinence incentives in the treatment of cocaine dependence. Findings for serotonergic medications tested in combination with CM have been mixed. In two trials with SSRI medications, one with fluoxetine (Schmitz et al., 1998) and one with citalopram (Moeller et al, 2007), positive treatment effects (medication vs. placebo) were observed when the background behavior therapy included an abstinence reinforcement intervention. However, an older study by Shoptaw et al. (2006) treating methamphetamine abusers failed to find a significant treatment interaction when abstinence-based incentives were combined with sertraline (50 mg bid). Further, a recent study by Winstanley et al. (2011) failed to find an interaction between fluoxetine and abstinence-contingent reinforcers in methadone maintained cocaine users. Thus, it appears that there may be some important pharmacological specificity related to drug actions on dopamine systems that supports the use of specific medications in combination with behavior therapies that rely on response to alternative positive reinforcement.

While the trials demonstrating a CM-medication interaction have been quite varied in the populations studied as well as the medications and methodologies employed, there is a striking consistency across them that supports the use of a dopaminergic medication for continued research on this interaction.

Further, although there are several possible explanations for this therapeutic interaction, the most intriguing and plausible is that the interaction occurs at a cellular level in dopaminergic brain systems that mediate response to rewarding stimuli such that dopamine levels are enhanced by the medication, which in turn allows the behavior therapy to be more efficacious than it otherwise would be. An exciting study proving additional support for this theory was recently published by Martinez et al. (2011). The study used PET imaging to examine dopamine signaling in the limbic striatum of cocaine users before and during the administration of a contingency management intervention. They found that both striatal dopamine receptor binding and presynaptic dopamine release were lower in volunteers who failed to respond to the abstinence reinforcement intervention. Thus, the study suggests that low dopamine transmission is associated with treatment failure and supports the hypothesis that a medication such as bupropion that increases striatial dopamine signaling may enhance response to a reward-based therapy.

Further support for a synergistic interaction between bupropion and abstinence incentives could significantly change clinical practice by offering a novel new paradigm for clinical treatment of stimulant abusers. Bupropion is the best candidate medication based on prior evidence for efficacy (Poling et al., 2006; Gray et al., 2010), recent positive findings from a laboratory drug versus money choice study by Stoops et al. (2012) and feasibility of use in clinical practice (e.g. low cost and low risk for significant side effects). The present proposed study will go well beyond previously published findings on this interaction to provide information that is both scientifically and clinically valuable. Specifically, the proposed study design is uniquely crafted to allow examination of patient-level mediators of the therapy interaction and to simultaneously answer two critically important clinical questions: 1) Does the medication help individuals with on-going stimulant use to stop that use initially? 2) Does the medication help with relapse prevention in those who have stopped stimulant use prior to medication exposure?

Overall, the proposed study is compelling because it conceptually differentiates the two key clinical issues in treatment of stimulant abusers- abstinence initiation and relapse prevention- and uses a design that efficiently and effectively tests a combined treatment approach on each. If successful, the study will point the way to a significant advance in improved treatment outcomes for this critical group of drug abusers.

4. Study Procedures

a. Study design, including the sequence and timing of study procedures (distinguish research procedures from those that are part of routine care).

Features of the study design are presented in detail below. All procedures including the study assessments, frequent urine testing schedule, and contingency management incentives will be conducted either at a methadone maintenance clinic site or at the BPRU with participants enrolled in methadone maintenance elsewhere. Background usual care consists of daily methadone dosing with medical management, and expectations of regular (usually once weekly) attendance at face to face individual and/or group counseling sessions.

b. Study duration and number of study visits required of research participants.

The study lasts for 30 weeks during which time participants are expected to report to their normal methadone clinic 7 days per week for methadone dosing (take home privileges, official holidays, and clinically determined take-home days excluded) and 3 times per week to study staff members either at REACH, Addiction Treatment Services, Glenwood Life Center, or at the BPRU for urine testing. Study visits include up to 3 screening visits (brief screen, full screen, medical exam), a baseline assessment, once weekly brief assessment visits (N = 20) and five longer assessment visits at weeks 6, 12, 18, 24, and 30.

c. Blinding, including justification for blinding or not blinding the trial, if applicable.

   Medication condition (active versus placebo bupropion) will be blinded to research and counseling staff who interact with participants in order to eliminate any potential biases from their interaction with participants.

d. Justification of why participants will not receive routine care or will have current therapy stopped.

   NA; participants will receive routine methadone maintenance.

e. Justification for inclusion of a placebo or non-treatment group.

   Placebo is included for scientific validity in a clinical trial needed to answer study questions related to medication efficacy.

f. Definition of treatment failure or participant removal criteria.

   There is no clear definition of treatment failure for this study sample of methadone maintained patients since long-term methadone maintenance is standard care for their chronic relapsing disorder. Patients could potentially be dismissed from treatment for violating clinic rules particularly those relating to violence, firearms possession, drug sales or or other unacceptable behavior at the clinic. Participants could be removed from the study if severe or persistent medication side-effects were observed or if new medical conditions developed including pregnancy or concomitant medications were started that made their continued participation unsafe.

g. Description of what happens to participants receiving therapy when study ends or if a participant's participation in the study ends prematurely.

   Study participants will continue on usual care methadone maintenance treatment at their normal methadone clinic after the study or in the event of premature withdrawal from the study.

Study Procedures: Urine Testing. Primary outcomes will be based on qualitative (pos/neg) urinalysis testing results using standard detection cut-off values. In order to detect recent use of cocaine, urine samples will be collected three times per week on non-consecutive days throughout the study, usually on Mon, Wed, and Fri. Sample collection will be observed if possible and all samples will additionally be tested for temperature and adulterants. A second sample will be requested if the original sample fails these tampering tests.

Study Procedures: CM Lead-In. Participants will enter the study following an intensive screening during which eligibility is determined, informed consent is signed and baseline assessment is completed. The study itself begins with a 6-week contingency management lead-in during which vouchers can be earned for submission of cocaine negative urine samples under an escalating schedule with reset to the original value in the case of missing or positive urines. A participant who stops cocaine use on the first day of the study and remains continuously cocaine abstinent would earn $145 by the end of week 6. However, many participants may start earning later than week 1 or experience resets and thus earn less.

Stratification and Random Assignment. A key feature of abstinence incentive programs is that participants tend to clearly dichotomize into responders and non-responders. The former initiate and sustain abstinence early in the program, while non-responders, either never initiate abstinence or do so intermittently or for only short periods of time before relapsing. In this study, participants will be stratified into CM responders versus non-responders based on submission of two consecutive weeks of cocaine-

free urines (6 samples) during study weeks 1- 6. Considerable experience with implementing these types of interventions suggests that there will be close to a 50/50 split between responders and non-responders by the end of the first month of the CM program.

By the end of study week 6, all participants still active in the study (and independent of exactly where they may be in the abstinence-contingent lead-In earnings plan) will be randomly assigned to bupropion vs placebo medication from within the responder vs non-responder strata and introduced to their tailored earnings schedule for the remainder of the study (see graphs below). Those who qualify for relapse prevention by submitting 2 weeks of negative urines will be randomized on the day they submit the 6th negative urine, while those who will clearly not qualify for relapse prevention will be randomized at the start of week 6.

Medication: Bupropion.

Commercially available sources of Bupropion XL and Microcrystalline Cellulose (placebo) will be obtained by the BPRU pharmacy. Qualified pharmacy staff will prepare double blind dosages by over-encapsulation of bupropion tablets. The inert substance in both active and placebo capsules will be Microcrystalline Cellulose. Active or placebo capsules will be placed in one-week blister pack kits compatible with daily dispensing requirements of the protocol. When a medication order is provided to the pharmacy, the blister packs will be labeled with a subject number and dispensed to the study site. Study medication blister-packs may be dispensed to participants by nursing staff at the methadone clinic dispensary window or by BPRU research staff. Each kit would contain an accountability log for clinic or study staff to document distribution of capsules from the kit to the study participant. Medication will be stored either in a secure vault at the methadone clinic site or in a pharmacy-approved safe within a locked room.

Medication dispensing will begin in study week 1 at which time all participants will begin receiving double blind capsules containing placebo. The advantage of this “placebo run-in” is that it stabilizes the medication taking regimen early in the study, precludes “placebo effects” at the time of random assignment and allows for a smooth transition into the post-random assignment study period without any change in study procedures. Participants may be randomized and potentially switched to active bupropion after giving their 6th cocaine negative urine following placebo initiation (week 3 randomization at the earliest) or at the start of study week 6 for those who provide urinalysis evidence of continued active drug use. Consistent with clinical practice for those randomized to active bupropion, there will be a brief (3-day) induction period followed by maintenance dosing at 300mg/day Medication dispensing will continue through week 30. Typically, only a one week supply will be given, but additional medication may be dispensed for holidays, weather-related or other emergencies.

Abstinence incentive schedule initially drug-negative.

The schematic below (left panel) depicts total weekly earnings for accelerating and decelerating phases for those who submit 6 or more negative urines during the CM run-in. The gradual escalation of voucher values begun in week 1 continues for a total of 12 weeks, which includes the Lead-in phase. Reset contingencies have been included for the relapse prevention participants to forestall early relapse. Once randomized to this condition, a cocaine positive or missing sample submitted prior to week 26 will result in reset to the $3 value. Following a reset, the participant will earn $3 then $3.50 for the first two cocaine negative samples then will return to the most recently achieved point in their earning schedule (e.g. $11 per sample) after the third consecutive cocaine negative sample. Extending the earning period from 24 to 26 weeks allows for completion of the entire earning schedule with 2 brief (single urine positive) slips during which an additional total of $13 could be earned.

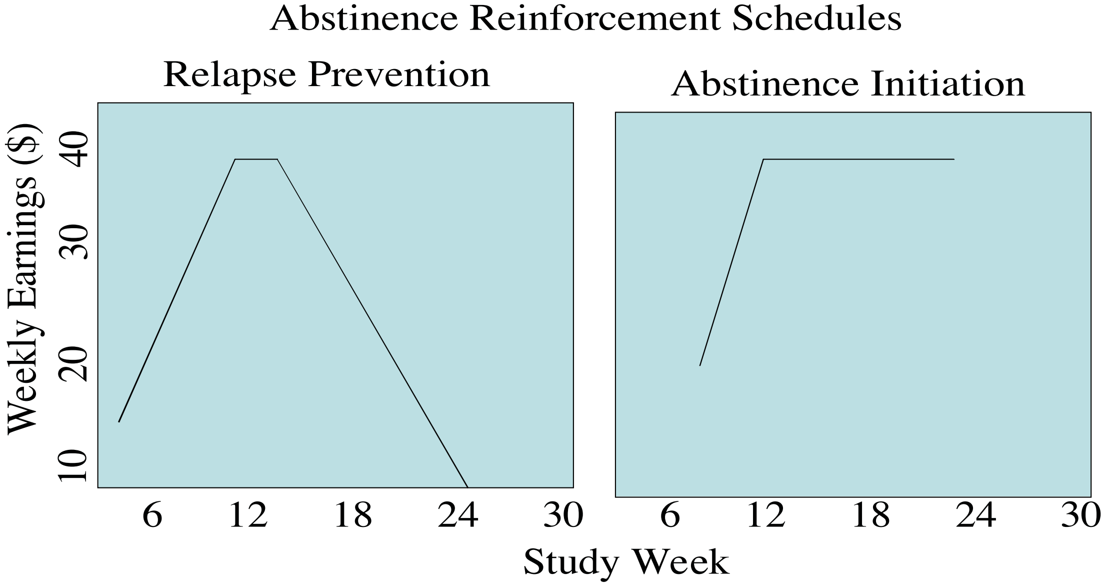

.

Following completion of the 10 week stable earning phase at $11 per negative urine, payment escalation reverses and the per urine payment schedule begins to decelerate. Note that the schedule is shown ending at study week 24. However, participants will be enrolled in the study for 30 weeks and allowed to continue earning through week 26. This allows time for those who may qualify later during Lead-In or have brief slips to complete the schedule and also time to track drug use and relapse measures after all reinforcement is withdrawn. Gradual pay reduction schedules have been used successfully in our clinical laboratory as relapse tests in cocaine self-administration (Donny et al., 2003, 2004) and smoking (Juliano et al., 2006) studies. Under this schedule, relapse will be delayed to the extent that the money alternative is perceived as more motivating and/or motivation for abstinence has shifted to reinforcers in the natural environment other than the monetary payments. Thus, bupropion is expected to slow or prevent relapse because the saliency of non-drug reinforcers maintaining abstinence will be enhanced. A maximum total of $688 can be earned by those who are continuously abstinent with the exception of no more than two single cocaine positive urines.

Abstinence incentive schedule: initially drug-positive.

The schematic above (right panel) depicts total weekly earnings for accelerating and decelerating phases for those who fail to reliably initiate abstinence during the initial 5 weeks. They will be randomly assigned at the start of study week 6 to begin taking double blind bupropion or placebo and will be able to earn abstinence incentives through week 26 of the trial while taking study medications. During week 5 they will be shown their new earnings schedule and encouraged to renew their efforts to become abstinent as soon as possible. The schedule under which they can earn abstinence-contingent vouchers has been adjusted so that total earnings for continuous abstinence are approximately equal to that of the initially abstinent relapse prevention group ($653 plus whatever might be earned during the lead-in phase). There will be a reset back to the original voucher value in this study to discourage relapse using the same rules described above. There is no decelerating payment phase as described for the Relapse Prevention group since the issue here is whether participants will initiate abstinence and for how long they will remain abstinent under a typical escalating schedule of payment.

Note that bonuses are included for both groups that are paid following submission of 3 consecutive cocaine negative urines. The number of bonuses that can be earned is fixed; once a bonus is dispersed it cannot be earned again following resets. It is important to note that there should be no biases developed among clinic patients as to which study earnings schedule is preferable since the total amount of money that can be earned during the study is comparable for those in the relapse prevention and abstinence initiation studies. Note, for example, that $114 is included in each schedule for bonus payments denoting achievement of 3 consecutive cocaine negative urines. Further, each schedule has features that may be perceived by participants as desirable (e.g. earnings escalation) and undesirable (declining values in

Relapse Prevention). Finally, participants will understand that they cannot choose, but will be assigned to their final earning schedule at or before study week 7. All these procedures circumvent any real or perceived inequities in the study stratification and assignment procedures.

Voucher redemption. Throughout the study, participants will be regularly updated on the amount of money in their voucher account. Once earned, voucher money is never taken away. Participants will be able to trade in vouchers at any time for a variety of tangible items and gift cards that will be kept on-site, to request that specific items be brought in for immediate trade-in or to save their voucher money for larger items that will be redeemed at a later time. The only restriction is on purchase of weapons, cigarettes or alcohol.

Contingency management summary. In addition to the schedules outlined above, participants will be reinforced for attendance of the short-assessment visit which occurs once-weekly. Participants can earn 1 bus token (good for a one-way trip) each time they attend and complete the short assessment. Participants may also receive other incentives for session attendance. The abstinence reinforcement schedules developed for this study are specially tailored to the study questions and subpopulations being addressed. Those who can stop their cocaine use before receiving medication may have higher dopamine function than those who do not (Martinez et al., 2011) but may still benefit from a dopamine-boosting medication for delaying or circumventing relapse. Their CM schedule, that incorporates a decelerating payment phase, is tailored for a time-to relapse assessment. In those who struggle to initiate abstinence, the escalating CM schedule will provide an appropriate background for detecting reinforcer boosting effects of a dopaminergic medication.

Study Measures (all but standard screening tools are appended in application)

Screening measures. Screening for stimulant and alcohol use disorders will be conducted using the DSM-5 checklist (American Psychiatric Association, 2013). Psychiatric screening will be conducted using the MINI International Neuropsychiatric Interview (Sheehan et al., 1998), a validated instrument that yields psychiatric diagnoses based on DSM-IV criteria. We will not administer the entire instrument. Rather, it will be used to screen for current major depression, mania/hypomania, psychosis, eating disorders and suicidality. We will continue to employ the Beck Depression Inventory as a measure of depressed mood and will conduct urine screen for cocaine and other drugs.

Weekly assessments include 3 X per week cocaine urine screen; periodic full drug screen, cocaine use self-report (7 day time line follow back), assessment of alcohol and tobacco use, the Cocaine Effects Scale visual analog measures of the pleasurable subjective effects of cocaine, and a depression symptom assessment.

Additional assessments administered at baseline and study weeks 6, 12, 18, 24, and 30:

Multiple Choice Drug vs Money scale (Griffiths et al., 1993) to assess relative reinforcement potency of cocaine; In this hypothetical task developed by Griffiths and colleagues (Griffiths et al., 1993), participants are presented with a series of choices between drug and money. The amount of drug remains constant across choices while the amount of money escalates. Amounts shown below incorporate the values of the actual per-urine payment schedule participants will later encounter (from $1.00 to $11) as well as values above and below this range. Outcome from the Multiple Choice Procedure is a “cross-over point”. This is the point at which the participant’s choice switches reliably from drug to money. The hypothesis is that those taking bupropion will switch their preference from drug to money at lower monetary values than will those taking placebo.

The Quick Inventory of Depressive Symptomatology (Rush et al., 2003) to further assess depression and depressive symptoms, including sleep and appetite changes which may also inform us about effects of cocaine withdrawal.

Snaith-Hamilton Pleasure Scale (Nakonezny et al., 2010) well suited for any population to assess pleasure derived from activities in the natural environment

Life Style Changes Scale (Silverman, 1998) to assess activities related to giving up drugs

Activities Assessment interview to probe in more depth for life style changes.

Health Risk Behavior Scale (Darke et al., 1991) to assess drug and sex-related HIV risk behaviors.

Fagerstrom Test of Nicotine Dependence (Heatherton et al., 1991) for assessment of current smoking dependence.

Physical status assessment of blood pressure; body weight.

Monetary-Choice Questionnaire to assess delay discounting (Epstein et al., 2003).

The Barratt Impulsiveness Scale to assess impulsiveness and impulsive personal traits (Patton et al., 1995).

Kirby Discounting Questionnaire

Neurocognitive assessment. A battery of tasks designed to measure neurocognitive performance has been developed at the BPRU (Mintzer and Stitzer, 2002) and will be adapted for this study. Functions to be tested include psychomotor speed/pattern recognition (e.g. digit symbol substitution), set shifting/conceptual flexibility (e.g.; trail making tests), divided attention, long-term/episodic memory (e.g. recognition and free recall) and short-term/working memory and focused attention (e.g. N-back task). The Conners Continuous Performance Test will also be used to assess performance in areas of inattentiveness, impulsivity, sustained attention, and vigilance (Epstein et al., 2002).

Inclusion/Exclusion Criteria

Inclusion Criteria:

- Over 18 years old
- Enrolled or meets federal requirements for enrollment in methadone maintenance treatment, and willing to visit the clinic daily
- Evidence of current cocaine dependence (DSM-5 criteria, self-report, urine test positive for cocaine during the intake process or by clinical record during the past 3 months).
- Willingness to swallow a capsule daily
- Willing to provide observed urine samples three times a week and participate in assessment procedures

Exclusion Criteria:

- Any history of epilepsy or seizure, including alcohol-, sedative-, or cocaine-related seizure;
- Any increased risk of seizure such as serious head trauma with a loss of consciousness of more than an hour duration, brain tumor, or other brain pathology increasing risk of seizure.
- Current eating disorder including anorexia or bulimia
- Current use (last 30 days) of medication that is contraindicated with bupropion or other bupropion products (e.g. antidepressants, antipsychotics, MAO-A inhibitors, theophyllines, systemic steroids, etc)
- Allergy to bupropion or budeprion
- Liver enzymes greater than 3x ULN
- Uncontrolled diabetes mellitus, or h/o diabetic coma
- Uncontrolled hypertension with BP > 140/90
- Current psychiatric diagnosis: schizophrenia, psychosis, major depression, mania, current suicidal ideation as determined by MINI psychiatric interview, cognitive impairment severe enough to preclude informed consent or valid responses on questionnaires
- Severe renal insufficiency (eGFR < 30 ml/min)
- Pregnancy or current breast feeding,
- Medical illness that in the view of the investigators would compromise participation in research, such as uncompensated congestive heart failure, recent history of myocardial infarction (<1year), or urologic conditions that inhibit urine collection.
- Advanced HIV infection requiring the use of HAART, or with CD4 T cell < 200/uL

5. **Drugs/ Substances/ Devices**
   a. The rationale for choosing the drug and dose or for choosing the device to be used.
   b. Justification and safety information if FDA approved drugs will be administered for non-FDA approved indications or if doses or routes of administration or participant populations are changed.
   c. Justification and safety information if non-FDA approved drugs without an IND will be administered.

Bupropion was selected as the best candidate medication for this trial based on prior evidence for its synergistic interaction with contingency management to enhance treatment outcomes for substance users (Poling et al., 2006; Gray et al., 2010), recent positive findings from a laboratory drug versus money choice study by Stoops et al. (2012) and feasibility of use in clinical practice including low cost and low risk for significant side effects. Dose selection (300 mg/day) is based on clinical guidelines, safety considerations and prior research in substance users. Selection of the slow release dosage form supports feasibility and compliance by allowing the majority of dose ingestions to be observed at the clinic.

We believe use of this medication is exempt from IND application requirements under CFR 312.2 as the study is not intended to provide data that would influence indications, labeling or advertising. Further, The investigation involves a standard dose (300mg/day) and route of administration (oral). Neither the dose, route of administration, study population or other factors significantly increases the risks (or acceptability of the risks) associated with use of this product.

6. **Study Statistics**
   a. Primary outcome variable.
   b. Secondary outcome variables.
   c. Statistical plan including sample size justification and interim data analysis.
   d. Early stopping rules.

Sample size and power.

The aim of this study is to evaluate whether bupropion will enhance cocaine abstinence among methadone-maintained patients who are abusing cocaine. This is being evaluated in a double-blind context. Further, based on evidence that individuals with cocaine use disorder typically present to the clinic with a urine sample that is positive for cocaine (which predicts continued use) or negative for cocaine (which predicts abstinence), we had originally proposed to treat those individuals as two separate groups and run 2 parallel studies (presented as Primary Aims 1 and 2), using contingency management as a background platform to support either abstinence or relapse prevention, respectively. Each aim was intended to enroll 100 individuals (N=200) total, to enable equal distribution of bupropion (n=50) and placebo (n=50) within each aim.

Based on unanticipated difficulties with recruitment, we are now collapsing the two parallel studies into the same overarching study, such that Primary Aim 1 will now be examining the effects of bupropion XL vs. placebo on 24-week abstinence outcomes during and after exposure to a behavioral intervention (CM). We are reducing our sample size to 100 individuals, which still preserves the required 50 participants per medication group that was identified in our power analyses as necessary to detect an effect of the medication. This approach will allow us to achieve our primary aim of determining whether bupropion will enhance cocaine abstinence despite challenges with recruitment and will enable this study to still provide clinically important information to advance the treatment of cocaine use disorder.

We are modifying our statistical plan to accommodate this change in sample size. We will retain the original primary outcome measure (biochemically-confirmed abstinence from cocaine), which will be assessed across all participants (independent of abstinence group) in the same statistical model. The endpoint will be binary (abstinent yes or no), and the treatment effect will be assessed using a repeated-measures logistic regression model having two main effects: medication and time, plus their interaction, as

well as any necessary covariates, including cocaine positive vs negative urine tests prior to random assignment as a covariate. Generalized linear mixed models (GLMM) will be used to analyze repeated measures data, since this technique can include participants with incomplete data without resorting to imputation. This approach has been found to minimize bias and maximize power in cases of incomplete follow-up (see Hedden et al, 2009). Data will then be examined using the nonbinary method described by McCann and Li (2012) in which participants who meet an end of study threshold of success (1 week EOS abstinence) are assigned values indicating their number of beyond threshold weeks of success (in our case 1-24). This distribution will be analyzed as a function of drug condition using a two-sided Vander Waerden two-sample test (Permutt and Berger, 2000).

All other analyses will be conducted in the overarching group that includes those abstinent and actively using at baseline. This approach will allow us to preserve all study Aims with a reduced sample size that will ensure the study can be completed in the time allowed.

The data analysis plan consists of the following:

1) Comparison of groups for baseline differences. Participants will be compared as a function of medication group on baseline characteristics to determine whether they are comparable with respect to general demographic features, baseline drug use, and reinforcement measures (drug vs. money relative reinforcing potency scale, Snaith-Hamilton Pleasure scale). Groups will be compared using t tests for continuous measures and chi-square tests for categorical characteristics. If medication groups are found to differ on baseline measures, then these characteristics will be used as covariates in subsequent analyses assessing treatment effect.

2) Primary outcome analyses: (see above)

3) Secondary outcome analyses: The main secondary outcome measure will be biochemically-confirmed abstinence from opiates. We hypothesize that participants receiving bupropion will have a higher proportion of opiate abstinent samples as well as for cocaine. Analyses conducted to assess the treatment effect for opiate use will be identical to those used for cocaine use (described above).

4) Other Secondary Outcome Analyses: Other secondary outcomes are reinforcement measures, such as the drug vs. money relative reinforcing potency scale, the Snaith-Hamilton Pleasure scale, as well as time engaging in non-drug-related activities and changes in neurocognitive performance. These measures will be collected at baseline and throughout the study. Analyses will be the same as those described for cocaine (GEE with medication group X time as factors). Participants receiving bupropion are hypothesized to find non-drug activities to be more reinforcing than participants receiving placebo, and to engage in more of them.

5) Exploratory mediation analysis. Measures collected at baseline including demographic characteristics, intake drug use severity, non-drug reinforcement, non-drug activities and neurocognitive performance will be examined separately to determine whether any predicts outcome independently of treatment. Next, we will add each of these measures to the models that assessed treatment effect, used for primary outcome analyses. If the treatment effect becomes diminished by addition of a third variable, this will be considered preliminary evidence that the third variable may mediate the effect of the treatment.

7. Risks

a. Medical risks, listing all procedures, their major and minor risks and expected frequency.
b. Steps taken to minimize the risks.
c. Plan for reporting unanticipated problems or study deviations.
d. Legal risks such as the risks that would be associated with breach of confidentiality.
e. Financial risks to the participants.

Bupropion XL is an FDA-approved antidepressant and smoking cessation pharmacotherapy and will be used in accordance with the prescription labeling instructions during this study. The extended release (XL) preparation allows for once-daily dosing and thus improved adherence. The medication will be titrated to an oral dose of 300 mg after 3 days at 150 mg daily. With a half-life of 21 hours, steady state is

reached after 8 days. Methadone maintenance is a long-standing and effective usual care treatment for opioid dependence but does not directly impact the concurrent use of stimulants alcohol or other drugs.

Risks associated with Bupropion XL side-effects

Major risks of bupropion are summarized below; a complete list of potential side-effects is included in the consent form.

Allergic reactions. Participants may experience allergic reactions to bupropion characterized by a rash, itching, swelling of the face, mouth, and throat. Allergic reactions are rare but can be life-threatening and can cause trouble breathing (anaphylactic reaction). Participants will be informed of this risk and instructed to stop taking the study medication and immediately contact medical staff if they experience any swelling or tingling in the mouth, develop a rash, or observe swelling, redness and/or peeling of the skin.

Seizures. The most relevant risk of bupropion is dose-dependent seizures, especially at doses higher than those proposed for this study (e.g. 450 mg daily). Bupropion is contra-indicated in patients with a seizure disorder, in those taking other medications containing bupropion, those taking monoamine oxidase A inhibitors (MOA-AI, e.g., phenelzine, risk of hypertensive crisis due to enhanced norepinephrine release), and those undergoing abrupt cessation of alcohol or sedative-hypnotic medications. These risks are managed by appropriate exclusion criteria and close medical monitoring described below.

Cardiovascular effects. While generally safe for use even in people with cardiovascular disease, review of the literature suggests that bupropion may increase blood pressure slightly.

Other side-effects and drug accumulation. Other side-effects associated with Bupropion XL include anxiety, agitation, insomnia, vivid or abnormal dreams, and weight loss. Less common side-effects include headache, hypertension, constipation, flatulence, nausea. All of these will be monitored during the study. Drug accumulation can occur in cases of severe liver disease and renal insufficiency. Patients with transaminases > 3 x ULN and an eGFR < 30 mL/min are excluded from participation.

Neuropsychiatric risks: The risk of suicidal thinking and behaviors, including completed suicide has been associated with antidepressants especially in children, adolescents, and young adults (< 25 yrs). The population targeted in this study may include young adults. While the incidence of neuropsychiatric symptoms among patients taking bupropion is of serious concern, it is expected to be rare. All participants will be closely monitored for any changes in mood, thought disturbance or agitation.

Potential drug-drug interactions: There is a general risk that Bupropion might have an adverse reaction with other medications, including methadone. The main causes of drug-drug interactions with bupropion are due to induction or inhibition of CYP2B6 dependent enzymatic pathways and potentiation of norepinephrine availability by MAO-A inhibitors.

Alcohol interaction. Bupropion may decrease tolerance for alcohol and high alcohol intake may enhance metabolism of bupropion through Interaction with CYP2B6 dependent enzymatic pathways. Drug interactions are also found with ritonavir, nelfinavir and efavirenz, Highly Active Antiretroviral Therapy drugs (HAART), with a reduction in the activity of HAART and/or a reduction in bupropion concentration (Robertson SM et al, 2008). Pre-clinical studies demonstrate acute exacerbation of bupropion toxicity by the non-selective MAOI phenelzine, most likely due to increase in norepinephrine release mediated by MAO-A. These potential interactions are handled by excluding participants taking HAART medications or MAO inhibitors and by monitoring alcohol intake.

Methadone interaction. Bupropion is unlikely to influence methadone metabolism: five studies of bupropion in methadone maintenance context showed no adverse reactions as a result of bupropion administration in conjunction with methadone and/or cocaine use (Poling et al., 2006 & 2007; Margolin et al., 1990 &1995, Levin FR et al., 2006).

Risks associated with methadone maintenance

This study will not be experimentally manipulating methadone doses and usual clinic policies and medical oversight will determine methadone doses. Methadone maintenance is associated with some risks including over sedation and QT prolongation. Any complaints of over-sedation will be evaluated by medical staff and methadone dose adjusted accordingly.

Other study-related risks

Diversion of study medication

Take-home doses of Bupropion XL will be provided for days when patients are given take-home (TH) methadone medication according to clinic policy. These will be for the most part single day TH on holidays, and for patients' unforeseeable emergencies. There is a small potential risk that study participants will divert or attempt to abuse the medication. However, bupropion is a Schedule V medication and is not associated with significant abuse liability. The limited take-home dosing and low abuse potential pose minimal risk of diversion.

Continued abuse of illicit drugs

Many participants who are enrolled in MM continue to abuse illicit drugs, and continued cocaine abuse is the targeted behavior of this study. Although there is no specific pharmacological risk of using cocaine and Bupropion concurrently, use of cocaine (or other illicit drugs) while enrolled in MM treatment is strongly discouraged and will be addressed by counselors during the study. Bupropion could affect the rate of cocaine and other drug use and this will be monitored as part of the study. Money or tangible goods earned in the contingency management program could supply funds for drug purchase. This is no different from the danger associated with funds secured in any other fashion. However, the process is self-limiting in contingency management since submission of a positive urine test immediately stops the dispersal of incentive payments.

Removal to controlled environment

Participants may experience a gap in research participation due to a period of non study-related hospitalization or incarceration. These individuals may be able to re-enter the research. However, such events will be handled on an individual basis according to best clinical judgment, and will take into account the safety and tolerability of inducting the participant back onto the study medication, the ability of the participant to resume reasonable compliance with the study requirements, and any marked changes in baseline cocaine use following the incident.

Pregnancy

There is a possibility that female participants may become pregnant while enrolled in the study and taking methadone and bupropion. Methadone is approved for use among pregnant women.while bupropion XL has been assigned to pregnancy category C where potential risks must be balanced by potential benefits in prescribing the medication. Urine pregnancy tests will be administered at 6-week intervals during the study as part of the health assessment. Study participation will be stopped for any woman who becomes pregnant.

Legal risks: Breach of confidentiality

Although staff members are highly trained to maintain participant confidentiality, there is always a risk that some of the confidential information collected could be revealed to people who are not involved in the research study. This could be embarrassing or risky to the participant and confidentiality breaches are considered to be very serious and are guarded against by standard precautions that include using only study ID's on CRF's and in data bases, using adequate password protection for data stored on computers and keeping identifying information (e.g. consent forms and PHI) in locked cabinets accessible only to research staff,

Financial risks. Participants who earn more than $600 in the study may have this income reported to the IRS (pending IRB approval of an exception for drug abuse-related research at the BPRU).

Risks Management

Risks inherent in exposure to the study medication, Bupropion XL, are managed first by application of appropriate study exclusion criteria that minimize risk, second by on-going in-person monitoring of study participants by methadone nursing and/or research staff and third by periodic administration of questionnaires that address changes in mood and suicidal ideations including assessment 1-2 weeks after starting study medication, which is a critical time for emergence of neuropsychiatric symptoms. Participants will be evaluated during the 6-week baseline phase of the protocol for excessive alcohol or sedative use. If medical staff judges that continued use of alcohol or sedatives is unsafe for interaction with bupropion, the individuals study participation will be terminated prior to randomization. Blood pressure will be monitored at baseline, 1-2 weeks after medication starts and at 6-week intervals

thereafter. Blood pressure criteria for medication adjustment or discontinuation would be as per clinical guidelines, i.e, BP > 140/90. Naturally, we will also be alert to any other cardiovascular adverse events as well with clinical judgment being used to determine dose adjustment or medication discontinuation.

In addition, study participants will receive periodic (e.g. every 6 weeks) assessment, for the presence of inter current health problems and for new concurrent medications that may pose a risk of drug-drug interactions. No specific laboratory monitoring is recommended for patients on bupropion. Participants will not be allowed to take any other antidepressants while they are on the study and will be discharged from the study if they need to begin taking any excluded medications that pose a risk of drug-drug interaction.

Experienced research, counseling, nursing, and medical staff will be readily available on-site as needed to assess and manage AE's and SAE's and research staff will be trained to report any responses indicating risk to medical and/or nursing personnel immediately for review and follow-up. Medical staff will evaluate participants who report clinically significant side effects possibly related to Bupropion (e.g., headache, agitation, insomnia) or who experience neuropsychiatric symptoms (e.g. depression, agitation). A code to break the drug blinds is available 24/7 if needed in response to a medical emergency. A participant may be put on close monitoring and/or referred for treatment if warranted in the judgment of medical staff. A participant may also be promptly withdrawn from the study protocol if medical staff indicates it is in the best interest of the patient. In all cases of persistent complaints, referral or study withdrawal, the case will be followed by medical staff until the complaint is resolved and the incident will be fully documented in study records and reported to IRB and NIDA.

Responsibility for monitoring data and safety during this clinical trial resides with the Principal Investigator (PI) Dr. Dunn and co-investigator Dr. Umbricht, M.D. AE's and SAE's are generally discovered through careful and frequent monitoring of participants by research and clinical staff using open-ended questions about how the participant has been feeling recently. Any complaint is probed for information about frequency, duration and severity, coded on a side-effects checklist and described in a written summary report. The Principal Investigator coordinates the preparation of semi-annual summary reports on the prevalence of side effects and adverse events observed during the course of the study. These reports will be provided annually to the local IRB and to the NIDA Program Official with the annual progress report. The PI will also be responsible for promptly notifying in writing the local Institutional Review Board if a serious adverse event (SAE) occurs. A SAE is defined as either: death; a life-threatening occurrence; hospitalization; new onset significant medical disorder; or significant exacerbation of an existing medical disorder.

In addition an independent panel of consultants will be contracted for bi-annual review of the risks-benefits ratio of the study. At mid-point during enrollment (when 100 randomized participants have completed the study) this panel will be provided with reports of adverse events and with participant outcome data (urine sample results) by unlabeled medication groups (A vs. B), as a function of study assignment (relapse prevention vs abstinence initiation). This information will be used to assess whether the risks reported are considered to be of unusual magnitude or frequency across any specific groups and to determine whether the study should be ended prematurely due to participant safety concerns. It is unlikely that such a small study would be interrupted because of an uneven distribution of adverse events experienced by any one group, however we will ensure that a qualified panel of reviewers makes this decision.

9. **Benefits**
   a. Description of the probable benefits for the participant and for society.

Study participation will directly benefit participants by providing them with methadone maintenance for opioid dependence, with increased urine monitoring beyond that available in usual care and with contingency management that is likely to reduce the amount of cocaine they use.

The study will provide important information about the efficacy of bupropion XL for reducing cocaine use in the context of methadone maintenance and background contingency management. The results of this study will provide valuable scientific information regarding the effects of dopamine systems activation on the saliency of non-drug monetary and non-monetary reinforcers as alternative to drug reinforcers. Study findings could also have direct clinical application for combined behavioral and pharmacological treatment of cocaine dependence should the hypotheses be borne out.

10. Payment and Remuneration

Detail compensation for participants including possible total compensation, proposed bonus, and any proposed reductions or penalties for not completing the protocol.

Each participant can earn $50 for screening, $210 for 6 longer assessment visits, and up to $688 in abstinence-contingent incentives based on cocaine negative urine screens for a total of $948 maximum earnings per participant. Participants can also earn Baltimore City Bus Tokens or be offered other incentives for attendance and/or other study related activities. There are no completion bonuses. Partial payment for study non-completers would be disbursed based on study assessments completed and contingent incentives earned.

11. Costs

a. Detail costs of study procedure(s) or drug (s) or substance(s) to participants and identify who will pay for them.

Participants may be required to pay treatment costs according to policies of the clinical performance site. All research-related costs including study medication and placebo, urine testing and abstinence incentive costs will be supported by the research grant. Any up-front fees associated with new participants entering ATS methadone treatment will be covered by the study.

Literature cited

American Psychiatric Association. (2013). Diagnostic and statistical manual of mental disorders (5th ed.). Arlington, VA: American Psychiatric Publishing.

Bisaga, A, Aharonovich, E, Cheng, WY, Levin, FR, Mariani, JJ, Raby, WN, Nunes, EV. A placebo-controlled trial of memantine for cocaine dependence with high-value voucher incentives during a pre-randomization lead-in period. Drug Alcohol Depend, 2010 111: 97-104. PMCID: PMC2930076

Darke S, Hall W, Heather N, Ward J, Wodak A. The reliability and validity of a secale to measure HIV risk-taking behaviour among intravenous drug users. AIDS, 1991; 5:181-185.

Donny EC, Bigelow GE, Walsh SL. Choosing to take cocaine in the human laboratory: Effects of cocaine dose, inter-choice interval, and magnitude of alternative reinforcement. Drug Alcohol Depend. 2003 69(3): 289-301.

Donny EC, Bigelow GE, Walsh SL. Assessing the initiation of cocaine self-administration in humans during abstinence: Effects of dose, alternative reinforcement, and priming. Psychopharmacology. 2004 172(3): 316-323.

Dutra, L, Stathopoulou, G, Basden, SL, Leyro, TM, Powers, MB, Otto, MW. A meta-analytic review of psychosocial interventions for substance use disorders. Am J Psychiatry. 2008 165: 179-87.

Elkashef, AM, Rawson, Ra, Anderson, AL, Li, SH, Holmes, T, Smith, EV, Chiang, N, Kahn, R, Vocci, F, Ling, W, Pearce, VJ, McCann, M, Campbell, J, Gorodetzky, C, Haning, W, Carlton, B, Mawhinney, J, Weiss, D (2008) Bupropion for the treatment of methamphetamine dependence.

Neuropsychopharmacology, 33, 1162-70.

Epstein JN, Erkanli A, Conners CK, Klaric J, Costello JE, Angold A. Relations between continuous performance test performance measures and ADHD behaviors. Journal of Abnormal Child Psychology. 2002; 31(5):543-554.

Epstein LH, Richards JB, Saad FG, Paluch RA, Roemmich JN, Lerman C. Comparison between two measures of delay discounting in smokers. Experimental and Clinical Psychopharmacology. 2003; 11(2):131-138.

Gray, KM, Carpenter, MJ, Baker, NL, Hartwell, KJ, Lewis, AL, Hiott, DW, Deas, D, Upadhyaya, HP. Bupropion SR and contingency management for adolescent smoking cessation. J Subst Abuse Treat. 2010 40: 77-86. PMCID: PMC2997899

Griffiths, RR, Troisi, JR, Silverman, K, Mumford, GK (1993) Multiple-choice procedure: an efficient approach for investigating drug reinforcement in humans. Behavioral Pharmacology, 4, 3-13.

Heatherton TF, Kozlowski LT, Frecker RC, Fagerström KO. The Fagerström Test for Nicotine Dependence: a revision of the Fagerström Tolerance Questionnaire. British Journal of Addiction (1991); 86:1119-1127.

Hedden, SL, Woolson, RF, Carter, RE, Palesch Y, Upadhyaya HP, Malcolm RJ. The impact of loss to follow-up on hypothesis tests of the treatment effect for several different statistical methods in substance abuse clinical trials. J Subst Abuse Treat. 2009 37: 54-63. PMCID: PMC2707817

Jones, HE, Johnson, RE, Bigelow, GE, Silverman, K, Mudric, T, Strain, EC. Safety and efficacy of l-tryptophan and behavioral incentives for treatment of cocaine dependence: A randomized clinical trial. Am J Addict. 2004 13: 421-37.

Juliano, L.M., Donny, E.C., Houtsmuller, E.J., & Stitzer, M.L. Experimental evidence for a casual relationship between smoking lapse and relapse. J Abnorm Psychol. 2006 115: 166-173.

Kosten, T, Oliveto, A, Feingold, A, Poling, J, Sevarino, K, McCance-Katz, E, Stine, S, Gonzalez, G, Gonsai, K . Desipramine and contingency management for cocaine and opiate dependence in buprenorphine maintained patients. Drug Alcohol Depend. 2003 70: 315-25.

Margolin, A, Kosten, Tr, Avants, K, Wilkins, J, Ling, W, Beckson, M, Arndt, IO, Cornish, J, Ascher, JA, Li, SH, Bridge, P. A multicenter trial of bupropion for cocaine dependence in methadone-maintained patients. Drug Alcohol Depend. 1995 40: 125-131.

Martinez, D, Carpenter, KM, Liu, F, Slifstein, M, Broft, A, Friedman, AC, Kumar, D, Heertum, RV, Kleber, HD, Nunes, E. Imaging dopamine transmission in cocaine dependence: Link between neurochemistry and response to treatment. Am J Psychiatry, 2011 168: 634-41.

McCann, DJ, Li, SH (2012) A novel, nonbinary evaluation of success and failure reveals bupropion efficacy vs methamphetamine dependence: Re-analysis of a multi site trial . CNS Neuroscience & Therapeutics, 18, 414-18.

Mintzer MZ, Stitzer ML. Cognitive impairment in methadone maintenance patients. Drug Alcohol Depend. 2002 Jun 1; 67(1): 41-51.

Moeller, FG, Schmitz, JM, Steinberg, JL, Green, CM, Reist, C, Lai, LY, Swann, AC, Grabowski, J. Citalopram combined with behavioral therapy reduces cocaine use: A double-blind, placebo-controlled trial. Am J Drug Alcohol Abuse. 2007 33: 367-78.

Nakonezny PA, Crmody TJ, Morris DW, Kurian BT, Trivedi MH. Psychometric evaluation of the Snaith-Hamilton pleasure scale in adult outpatients with major depressive disorder. Int Clin Psychopharmacol. 2010 25: 328-333. PMCID: PMC2957191

Patton JH, Stanford MS, Barratt ES. Factor structure of the barratt impulsiveness scale. Journal of Clinical Psychology. 1995; 51(6)768-774.

Peirce JM, Petry NM, Stitzer ML, Blaine J, Kellogg S, Satterfield F, Schwartz M, Kraznansky J, Pencer E, Silva-Vasquez L, Kirby KC, Royer-Malvestuto C, Roll JM, Cohen A, Copersino ML, Kolodner K, Li R. Effects of lower-cost incentives on stimulant abstinence in methadone maintenance treatment. Arch Gen Psychiatry. 2006 63: 201-208.

Petry NM, Peirce JM, Stitzer ML, Blaine J, Roll JM, Cohen A, Obert J, Killeen T, Saladin ME, Cowell M, Kirby KC, Sterling R, Royer-Malvestuto C, Hamilton J, Booth RE, Macdonald M, Liebert M, Rader L, Burns R, DiMaria J, Copersino M, Stabile PQ, Kolodner K, Li R. Effect of prize-based incentives on outcomes in stimulant abusers in outpatient psychosocial treatment programs: a national drug abuse treatment clinical trials network study. Arch Gen Psychiatry. 2005 62: 1148-1156.

Poling J, Oliveto A, Petry N, Sofuoglu M, Gonsai K, Gonzalez G, Martell B, Kosten TR. Six-month trial of bupropion with contingency management for cocaine dependence in a methadone-maintained population. Arch Gen Psychiatry. 2006 Feb; 63(2): 219-228.

Poling J, Pruzinsky R, Kosten TR, Gonsai K, Sofuoglu M, Gonzalez G, Oliveto A. Clinical efficacy of citalopram alone or augmented with bupropion in methadone-stabilized patients. Am J Addict. 2007 May-Jun; 16(3): 187-194.

Rush, A. J., Trivedi, M. H., Ibrahim, H. M., Carmody, T. J., Arnow, B., Klein, D. N., et al. (2003). The 16-item quick inventory of depressive symptomatology (QIDS), clinician rating (QIDS–C), and self-report (QIDS–SR): A psychometric evaluation in patients with chronic major depression. Biological Psychiatry, 54(5), 573–583.

Sheehan, D et al. (1998) the MINI-International Neuropsychiatric Interview (M.I.N.I.): Development and validation of a structured diagnostic psychiatric interview for DSM-IV and ICD-10. Journal of Clinical Psychiatry, 59, suppl 20: 22-33.

Schmitz, JM, Mooney, ME, Moeller, FG, Stotts, AL, Green, C, Grabowski, J. Levodopa pharmacotherapy for cocaine dependence: Choosing the optimal behavioral therapy platform. Drug Alcohol Depend. 2008 94: 142-50. PMCID: PMC2293271

Schmitz JM, Rhoades HM, Elk R, Creson D, Hussein I, Grabowski J. Medication take-home doses and contingency management. Exp Clin Psychopharmacol. 1998 6: 162-68.

Shoptaw S, Heinzerling KG, Rotheram-Fuller E, Kao UH, Wang PC, Bholat MA, Ling W. Bupropion hydrochloide versus placebo in combination with cognitive behavioral therapy for the treatment of cocaine abuse/dependence. J Addict Dis. 2008a 27: 13-23.

Shoptaw S, Heinzerling KG, Rotheram-Fuller E, Steward T, Wang J, Swanson AN, De La Garza R, Newton T. Ling W. Randomized, placebo-controlled trial of bupropion for the treatment of methamphetamine dependence. Drug Alcohol Depend. 2008b 96: 222-232.

Shoptaw S, Huber A, Peck J, Yang X, Liu J, Dang J, Roll J, Shapiro B, Rotheram-Fuller E, Ling W. Randomized placebo-controlled trial of sertraline and contingency management for the treatment of methamphetamine dependence. Drug Alcohol Depend. 2006 85: 12-18.

Silverman K, Higgins ST, Brooner R, Montoya ID, Cone EJ, Schuster CR, Preston KL. Sustained cocaine

abstinence in methadone maintenance patients through voucher-based reinforcement therapy. Arch Gen Psychiatry. 1996 53: 409-415.

Silverman K, Robles E, Mudric T, Bigelow GE, Stitzer ML. A randomized trial of long-term reinforcement of cocaine abstinence in methadone –maintained patients who inject drugs. J Consult Clin Psychol. 2004 72: 839-54.

Silverman K, Wong CJ, Umbricht-Schneiter A, Montoya ID, Schuster CR, Preston KL. Broad beneficial effects of cocaine abstinence reinforcement among methadone patients. J Consult Clin Psychol. 1998 66: 811-24.

Stoops, WW, Lile, JA, Glaser, PE, Hays, LR, Rush, CR (2012) Influence of acute bupropion pre-treatment on the effects of intranasal cocaine. Addiction, 107, 1140-47.

Stitzer, M.L. and Walsh, S.L. (1997) Psychostimulant abuse: The case for combined behavioral and pharmacological treatments. Pharmacology, Biochemistry & Behavior, 57, 457-470.

Winstanley EL, Bigelow GE, Silverman K, Johnson RE, Strain EC. A randomized controlled trial of fluoxetine in the treatment of cocaine dependence among methadone-maintained patients. J Subst Abuse Treat. 2011 40(3): 255-264. PMCID: PMC3078567
