# Supplementary material for: Bupropion Slow Release vs Placebo With Adaptive Incentives for Cocaine Use Disorder in Persons Receiving Methadone for Opioid Use Disorder: A Randomized Clinical Trial
Source: JAMA Netw Open. 2023 Mar 15;6(3):e232278. doi: 10.1001/jamanetworkopen.2023.2278 (PMC10018324; doi:10.1001/jamanetworkopen.2023.2278)
Supplement: Supplement 2. — eTable 1. Study Inclusion Criteria eTable 2. Incentive Structure eTable 3. MITT Participant Demographics eTable 4. Adverse Events [file jamanetwopen-e232278-s002.pdf]

Supplemental Online Content

Ware OD, Sweeney MM, Cunningham C, Umbricht A, Stitzer M, Dunn KE. Bupropion slow release vs placebo with adaptive incentives for cocaine use disorder in persons receiving methadone for opioid use disorder: a randomized clinical trial. *JAMA Netw Open*. 2023;6(3):e232278.
doi:10.1001/jamanetworkopen.2023.2278

eTable 1. Study Inclusion Criteria
eTable 2. Incentive Structure
eTable 3. MITT Participant Demographics
eTable 4. Adverse Events

This supplemental material has been provided by the authors to give readers additional information about their work.

© 2023 Ware OD et al. *JAMA Network Open.*

eTable 1. Study Inclusion Criteria

Inclusion Criteria:

1. Over 18 years old
2. Enrolled or meets federal requirements for enrollment in methadone maintenance treatment, and willing to visit the clinic daily
3. Evidence of current cocaine dependence (DSM-5 criteria, self-report, urine test positive for cocaine during the intake process or by clinical record during the past 3 months).
4. Willingness to swallow a capsule daily
5. Willing to provide observed urine samples three times a week and participate in assessment procedures

Exclusion Criteria:

1. Any history of epilepsy or seizure, including alcohol-, sedative-, or cocaine-related seizure;
2. Any increased risk of seizure such as serious head trauma with a loss of consciousness of more than an hour duration, brain tumor, or other brain pathology increasing risk of seizure.
3. Current eating disorder including anorexia or bulimia
4. Current use (last 30 days) of medication that is contraindicated with bupropion or other bupropion products (e.g. antidepressants, antipsychotics, MAO-A inhibitors, theophyllines, systemic steroids, etc)
5. Allergy to bupropion or budeprion
6. Liver enzymes greater than 3x ULN
7. Uncontrolled diabetes mellitus, or h/o diabetic coma
8. Uncontrolled hypertension with BP > 140/90
9. Current psychiatric diagnosis: schizophrenia, psychosis, major depression, mania, current suicidal ideation as determined by MINI psychiatric interview, cognitive impairment severe enough to preclude informed consent or valid responses on questionnaires
10. Severe renal insufficiency (eGFR < 30 ml/min)
11. Pregnancy or current breast feeding,
12. Medical illness that in the view of the investigators would compromise participation in research, such as uncompensated congestive heart failure, recent history of myocardial infarction (<1year), or urologic conditions that inhibit urine collection.
13. Advanced HIV infection requiring the use of HAART, or with CD4 T cell < 200/uL

© 2023 Ware OD et al. *JAMA Network Open.*

eTable 2. Incentive Structure

Study Week	Consecutive Visit	Incentive Induction ($)	Abstinence Initiation ($)	Relapse Prevention ($)	Bonuses ($)
1	1	0.5	-	-	-
1	2	1.0	-	-	-
1	3	1.5	-	-	-
2	4	2.0	-	-	-
2	5	2.5	-	-	-
2	6	3.0	-	-	-
3	7	3.5	-	-	-
3	8	4.0	-	-	-
3	9	4.5	-	-	-
4	10	5.0	-	-	-
4	11	5.5	-	-	-
4	12	6.0	-	-	-
5	13	6.5	-	-	-
5	14	7.0	-	-	-
5	15	7.5	-	-	-
6	16	8.0	-	-	-
6	17	8.5	-	-	-
6	18	9.0	-	-	-
7	19		3.0	9.5	0
7	20		3.5	10.0	0
7	21		4.0	10.5	10
8	22		4.5	11	0
8	23		5.0	11	0
8	24		5.5	11	10
9	25		6.0	11	0
9	26		6.5	11	0
9	27		7.0	11	10
10	28		7.5	11	0
10	29		8.0	11	0
10	30		8.5	11	10
11	31		9.0	11	0
11	32		9.5	11	0
11	33		10.0	11	10
12	34		10.5	11	0
12	35		11	11	0
12	36		11	11	10
13	37		11	11	0
13	38		11	11	0
13	39		11	11	10
14	40		11	11	0
14	41		11	11	0

© 2023 Ware OD et al. *JAMA Network Open.*

14	42		11	11	7
15	43		11	11	0
15	44		11	11	0
15	45		11	11	7
16	46		11	11	0
16	47		11	11	0
16	48		11	11	7
17	49		11	11	0
17	50		11	11	0
17	51		11	11	7
18	52		11	10.5	0
18	53		11	10.0	0
18	54		11	9.5	7
19	55		11	9.0	0
19	56		11	8.5	0
19	57		11	8.0	3
20	58		11	7.5	0
20	59		11	7.0	0
20	60		11	6.5	3
21	61		11	6.0	0
21	62		11	5.5	0
21	63		11	5.0	3
22	64		11	4.5	0
22	65		11	4.0	0
22	66		11	3.5	0
23	67		11	3.0	0
23	68		11	2.5	0
23	69		11	2.0	0
24	70		11	1.5	0
24	71		11	1.0	0
24	72		11	0.5	0
		85.50	526.00	475.50	114.00

All participants received same incentive conditions in weeks 1-6. Assignment to Relapse Prevention (RP) determined by achieving 6 consecutive negative samples during weeks 1-6 (RP). All other participants assigned to Abstinence Initiation (AI).

Bonuses based on number of consecutive abstinent samples and were available on any study days once the bonus requirement was met. All participants were eligible to earn seven $10, five $7, and three $3 bonuses and values decreased based on number earned rather than study week.

Values presented in dollars. All incentives ended in week 24

© 2023 Ware OD et al. *JAMA Network Open.*

eTable 3. MITT Participant Demographics

	Total (N=80) N (%)	Medication Group			Incentive Condition			Completion Status		
		Bupropion-SR (N=40)	Placebo (N=40)	p-value	Abstinence Initiation (N=60)	Relapse Prevention (N=20)	p-value	Noncompleters (N=28)	Completers (N=52)	p-value
Demographic Characteristics										
Age (years, mean [SD])	46.0 (9.4)	44.5 (10.8)	47.5 (7.9)	0.16	45.7 (9.9)	47.1 (8.3)	0.56	45.0 (1.6)	46.6 (10.2)	0.50
Male (%)	52 (65.0)	25 (62.5)	27 (67.5)	0.82	21 (35.0)	7 (35.0)	1.00	7 (25.0)	30 (59.6)	
Race[a] (%)				0.65			0.20			0.12
White/Caucasian	35 (43.8)	19 (47.5)	27 (40.0)		29 (48.3)	6 (30.0)		17 (60.7)	18 (34.6)	
Black/African American	42 (52.5)	20 (50.0)	22 (55.0)		29 (48.3)	13 (65.0)		11 (39.3)	31 (59.6)	
American Indian	1 (1.3)	0.0	1 (2.5)		0.0	1 (5.0)		0	1 (1.9)	
More than once race	2 (2.5)	1 (2.5)	1 (2.5)		1 (3.3)	0.0		0	2 (3.8)	
Hispanic Ethnicity[a] (%)	3 (3.8)	1 (2.5)	2 (5.0)	1.00	1 (3.3)	1 (5.0)	1.00	0	3 (5.8)	0.54
Drug Use Characteristics										
Methadone Treatment <1 year (%)	32 (40)	19 (47.5)	13 (32.5)		25 (41.7)	7 (35.0)		16 (57.1)	16 (30.8)	
Methadone Dose (mgs, mean [SD])	87.8 (29.4)	86.4 (28.1)	89.1 (30.6)	0.68	86.7 (29.5)	90.8 (28.9)	0.59	76.6 (4.3)	93.7 (30.6)	
Injection Drug Use (past 30 days, %)	19 (23.8)	13 (32.5)	6 (15.0)	0.11	14 (23.3)	5 (25.0)	1.00	6 (21.4)	13 (25.0)	0.79
Cocaine Positive Urine Sample (%)	59 (73.8)	31 (77.5)	28 (70.0)	0.61	52 (86.7)	7 (35.0)	**<0.001**	23 (82.1)	36 (69.2)	0.29
Cocaine Use Disorder										
Severity (range 0-11, mean [SD])	7.6 (2.6)	7.4 (2.4)	7.8 (2.7)	0.49	7.6 (2.6)	7.6 (2.5)	0.98	7.1 (2.4)	7.9 (2.6)	0.19
Mild (%)	7 (8.8)	3 (7.5)	4 (10.0)	1.00	6 (10.0)	1 (5.0)	0.67	3 (10.7)	4 (7.7)	0.68
Moderate (%)	10 (12.5)	4 (10.0)	6 (15.0)	0.74	7 (11.7)	3 (15.0)	0.70	3 (10.7)	7 (13.5)	0.69
Severe (%)	63 (78.8)	33 (82.5)	30 (75.0)	0.59	47 (78.3)	16 (80.0)	1.00	22 (78.6)	41 (78.9)	1.00
Alcohol Use Disorder (%)	8 (10.0)	3 (7.5)	15 (12.5)	0.71	4 (6.7)	4 (20.0)	0.10	1 (3.5)	7 (13.5)	1.00
Severity (range 0-11, mean [SD])	0.77 (2.1)	0.60 (1.8)	0.93 (2.3)	0.48	0.5 (1.6)	1.6 (3.0)	**0.05**	0.3 (1.2)	1.1 (2.4)	0.13
Cigarette Smoker (%)	72 (90.0)	35 (87.5)	92.5	0.71	53 (88.3)	19 (95.0)	0.67	25 (89.2)	47 (90.4)	1.00

mITT= modified intention-to-treat (N=80), defined as randomized participants taking ≥1 dose of study medication
mgs= milligrams, SD=standard deviation, SR= slow release
P-values represent comparisons within groups based upon Chi-squares for dichotomous and independent groups t-tests for continuous variables
a. Race and ethnicity was self-defined by participants on patient-reported measure intended to characterize the sample

eTable 4. Adverse Events

Adverse Event	Number of Participants		Number of Adverse Events	
	Bupropion-SR (N=40)	Placebo (N=40)	Bupropion-SR (N=40)	Placebo (N=40)
Abdominal Cramp	2	0	2	0
Abdominal Pain	0	1	1	1
Chills	1	0	1	0
Depression	3	1	5	1
Dizziness	1	0	1	0
Drowsiness	1	0	1	0
Gastro Esophageal Reflux Disease (GERD)	0	1	0	1
Headache	3	1	3	3
Hypertension	1	0	1	0
Pain Lower Extremity	0	1	0	1
Pain Lower Extremity	0	1	0	1
Skin Rash	1	1	1	1
Stomach Cramps	1	0	1	0
Stomach Pain	0	1	0	1
Sweat increased	1	0	1	0
Tinnitus	1	0	1	0
Vomiting	1	1	1	1

Events collapsed across mild, moderate, and severe and rated as being possibly, probably, or definitely related to study medication.

© 2023 Ware OD et al. *JAMA Network Open.*
