# Supplementary material for: Bupropion Slow Release vs Placebo With Adaptive Incentives for Cocaine Use Disorder in Persons Receiving Methadone for Opioid Use Disorder: A Randomized Clinical Trial
Source: JAMA Netw Open. 2023 Mar 15;6(3):e232278. doi: 10.1001/jamanetworkopen.2023.2278 (PMC10018324; doi:10.1001/jamanetworkopen.2023.2278)
Supplement: Supplement 3. — Data Sharing Statement [file jamanetwopen-e232278-s003.pdf]

Data Sharing Statement

Ware. Bupropion Slow Release vs Placebo With Adaptive Incentives for Cocaine Use Disorder in Persons Receiving Methadone for Opioid Use Disorder: A Randomized Clinical Trial. *JAMA Netw Open*. Published online March 15, 2023. doi:10.1001/jamanetworkopen.2023.2278

Data
Data available: Yes
Data types: Deidentified participant data
How to access data: de-identified data can be made available upon request via a data-transfer agreement.
When available: With publication

Supporting Documents
Document types: Informed consent form, Other (please specify)
Additional Information: protocol
How to access documents: documents will be available upon request
When available: With publication

Additional Information
Who can access the data: Data will be available to persons who initiate a data transfer agreement
Types of analyses: Secondary analyses, meta-analyses, reanalyses of primary outcomes
Mechanisms of data availability: after approval and completion of data-transfer agreement

© 2023 Ware OD et al. *JAMA Network Open*.
